# Human iPSC-derived *APOE4/4* Alzheimer´s disease astrocytes exhibit a senescent and pro-inflammatory state that compromises neuronal support

**DOI:** 10.1186/s12974-025-03607-z

**Published:** 2025-12-12

**Authors:** Laura Caceres-Palomo, Elisabeth Sanchez-Mejias, Laura Trujillo-Estrada, Juan Jose Perez-Moreno, Elba Lopez-Oliva, Tau En Lim, Leah DeFlitch, Serena H. Chang, Lucas Kampman, M. Ryan Corces, Mathew Blurton-Jones, Ines Moreno-Gonzalez, Alberto Pascual, Javier Vitorica, Juan Antonio Garcia-Leon, Antonia Gutierrez

**Affiliations:** 1 Dpto. Biologia Celular, Genetica y Fisiologia, Instituto de Investigacion Biomedica de Malaga-IBIMA-Plataforma Bionand, Facultad de Ciencias, Universidad de Malaga, Malaga, 29071 Spain; 2 Centro de Investigacion Biomedica en Red sobre Enfermedades Neurodegenerativas (CIBERNED), Madrid, Spain; 3 Instituto de Biomedicina de Sevilla (IBiS)-Hospital Universitario Virgen del Rocio, CSIC/Universidad de Sevilla, Sevilla, 41013 Spain; 4 Departamento of Biologia Celular, Facultad de Biologia, Universidad de Sevilla, Sevilla, Spain; 5 Institute for Memory Impairments and Neurological Disorders, University of California, Irvine, Irvine, CA 92697 USA; 6 Gladstone Institute of Neurological Disease, Gladstone Institutes, San Francisco, CA USA; 7 Neuroscience Graduate Program, University of California San Francisco, San Francisco, CA USA; 8 Gladstone Institute of Data Science and Biotechnology, Gladstone Institutes, San Francisco, CA USA; 9 Department of Neurology, University of California San Francisco, San Francisco, CA USA; 10 Present address: Basic Sciences Division and Computational Biology Program and Molecular and Cellular Biology Graduate Program, Fred Hutchinson Cancer Center, University of Washington, Seattle, WA USA; 11 Department of Neurobiology & Behavior, University of California, Irvine, CA 92697 USA; 12 Dpto. Bioquimica y Biologia Molecular, Facultad de Farmacia, Universidad de Sevilla, Sevilla, 41012 Spain

**Keywords:** Glial dysfunction, Astrocytes, APOE, Cellular model, Stem cells, Neurodegeneration, Alzheimer´s disease, Senescence, Neuroinflammation

## Abstract

Alzheimer´s disease (AD) is dominated by a complex cellular pathology which involves most brain cell types with glial cells increasingly recognized as playing fundamental roles in neurodegeneration. Astrocytes, which perform essential functions in preserving brain homeostasis, present a reactive phenotype in the AD brains with still unknown consequences. In this study, we generated and characterized human induced pluripotent stem cell (hiPSC)-derived astrocytes from AD patients harboring the *APOE4/4* genotype, the greatest genetic risk factor for late-onset AD. Disease astrocytes showed a reactive phenotype. In addition, they showed altered mitochondrial network including perinuclear clustering of mitochondria, enhanced mitochondrial fusion and higher production of reactive oxygen species which, unexpectedly, were coincident with increased oxidative phosphorylation and glycolysis. As these mitochondrial features are related to the acquisition of cell senescence, we evaluated this at the transcriptome level and found that these AD-derived astrocytes significantly upregulated gene signatures of cellular senescence and displayed a senescence-associated secretory phenotype (SASP). To verify this finding, we observed senescence-related DNA damage response in a significant proportion of cells in the cerebral cortex of AD patients, with most of these cells being astrocytes. Finally, we confirmed that this astrocytic senescent and proinflammatory phenotype is associated with a reduced neuronal support, evidencing that *APOE4/4* AD astrocytes present intrinsic features that may compromise brain homeostasis and promote neurodegeneration. Addressing the causes and consequences of this astrocytic dysfunctionality should help to elucidate novel therapeutic targets able to modify the neurodegeneration present in AD.

**Supplementary Information:**

The online version contains supplementary material available at 10.1186/s12974-025-03607-z.

## Background

Alzheimer´s disease (AD) is a highly debilitating and fatal neurodegenerative condition affecting the elderly population and for which, to date, there is no known cure. AD accounts for most of dementia cases (currently 50 million worldwide, which will triple in the coming decades), representing a huge emotional and economic impact on society [1]. Recently, few passive immunotherapies directed towards amyloid-beta (Ab) have been approved by the FDA [2] which, despite being effective in eliminating Ab accumulation from AD brains, only produce a moderate clinical beneficial effect in a subgroup of patients, reinforcing the accepted idea that multiple amyloid-independent mechanisms are dysregulated in the AD brains [3]. In fact, most if not all brain cell types are affected during the progression of AD [4], evidencing that elucidating the different cell-specific mechanisms contributing to disease progression is mandatory to be able to significantly improve the disease course and, therefore, the quality of life of AD patients and their families.

Over the last decades, glial cells have been framed as key pieces in the AD pathology puzzle [5] with microglia, the main innate immune cells in the brain parenchyma, having a predominant and causal role in the pathological process [6]. These brain resident myeloid cells exhibit diverse molecular and functional activation states depending on-disease stage and brain region, with protective or harmful effects [7]. In addition, differences in microglial responses have been found between human and murine models of disease [8–10]. On the other hand, astroglial reactivity is also a long reported feature present in AD brains which functional consequences are still unknown [11], as astrocytes play critical roles in supporting neuronal functions [12]. In the AD brain, astrocytes present a particular transcriptomic profile characterized by the loss of homeostatic genes and the acquisition of a reactive state [13, 14]. In addition, the presence of several pathological phenotypes of astrocytes has been recently identified in murine models and in AD brains [15].

Most of the research performed in the AD field in the last three decades has employed murine transgenic animal models harboring familial AD-associated mutations, which has provided invaluable information about disease pathology, but this knowledge has not been translated into effective therapies, most likely due to inherent biological differences between mice and humans. Meanwhile neuronal subpopulations and functioning are relatively well-conserved between mice and humans, important differences exist in the case of astrocytes. Hominid astrocytes are significantly larger and more complex than murine ones, with faster intracellular calcium activity and a higher variability of subtypes [16]. They present greater susceptibility to oxidative stress than mouse astrocytes, due to differences in mitochondrial physiology and detoxification pathways and differential inflammatory responses to insults [17]. Therefore, it is tentative to state that human-derived astrocytes must be used to address their role in human pathology as AD. On the other hand, *APOE4* genotype is the major genetic risk factor for developing sporadic late-onset AD [18], and carrying two copies of this allele may be even a causative factor contributing to early-onset forms of the disease [19] and, if so, the most common genetic cause of AD. APOE is primarily expressed by astrocytes in the brain under physiological conditions, and has a key role in the bioenergetic homeostasis of the brain. The contribution of astrocytes and their APOE variants on AD remains to be elucidated.

In the present study we generated human induced pluripotent stem cell (hiPSC)-derived astrocytes from AD patients harboring the *APOE4/4* genotype and found that they show intrinsic functional alterations in basal conditions including a reactive phenotype. Mitochondrial network was significantly altered in disease astrocytes, exhibiting a preferential perinuclear location, an upregulation of mitochondrial fusion, co-localization with autophagosomes and higher production of mitochondrial ROS, together with an increased oxidative phosphorylation and glycolysis. These disease-related features coincided with the acquisition of a senescent state as RNA sequencing (RNA-seq) and gene set enrichment analysis revealed upregulation of senescence transcriptional programs. In addition, these AD-derived astrocytes also displayed a senescence-associated secretory phenotype (SASP), which was exacerbated by the stimulation of these astrocytes with pro-inflammatory cytokines. Noteworthy, in the cerebral cortex of AD patients we observed a significant proportion of cells with senescence-related DNA damage response, the majority of which were astrocytes. Finally, we confirmed that this phenotype was associated with deficient neuronal support, evidencing that AD astrocytes may contribute to neurodegeneration.

## Materials and methods

### Human iPSCs lines and culture conditions

Human iPSC lines were generated by the University of California, Irvine Alzheimer’s Disease Research Center (UCI ADRC) Induced Pluripotent Stem Cell Core under approved Institutional Review Boards (IRB) and human Stem Cell Research Oversight (hSCRO) committee protocols. Reprogramming was performed using non integrating Sendai virus (CytoTune-iPS 2.0, Thermo Fischer). Normal 46XX or 46XY karyotypes were confirmed by Microarray-based Comparative Genomic Hybridization (aCGH, Cell Line Genetics). Pluripotency was verified by trilineage differentiation (STEMdiff trilineage kit, Stem Cell Technologies), and sterility confirmed by MycoAlert (Lonza). The following iPSCs lines were used in this study: three iPSCs lines derived from AD patients (*N* = 3, 74 ± 3.46 years) with *APOE4/4* genotype (lines hiPSCs 20, 23 and 96) and three iPSCs lines from normal cognition individuals without AD (*N* = 3, 80.67 ± 2.08 years) and with *APOE3/3* genotype (lines hiPSCs 76, 77 and 107). iPSCs were maintained in feeder free conditions using mTeSR plus medium (StemCell Technologies, 100–0276) on Geltrex-coated plates (Thermo Fisher Scientific, A1413201) and splitting twice a week using Versene (Lonza, 17161E). The experimentation with human iPSCs was approved by the ethical committees of the University of Malaga (CEUMA: 101–2018-T) and the Spanish Instituto de Salud Carlos III (requests 491_396_1 and 500_405_1).

### Differentiation of neural precursor cells (NPCs) and astrocytes from human iPSCs

The protocols used were based on the ones described by Perriot et al. [20] with small adaptations. Briefly, iPSCs were dissociated into single cells using accutase (Sigma-Aldrich, A6964) and seeded at 100–200,000 cells/cm^2^ on Geltrex with mTeSR plus medium containing 1:100 of RevitaCell (Thermo Fisher Scientific, A2644501). Next day (day 0), when cells reached 100% confluence, medium was switched to neural induction medium (see supplementary (Supp.) information for detailed media composition) for 12 days, changing medium every day. At this time, neural rosettes should appear in the culture, and they were dissociated 3 times with dispase (Sigma-Aldrich, D4693), when reached confluence, in rosettes expansion medium. Since this moment, medium was changed partially every 2 days. After this phase, approximately 20 days after the beginning of differentiation, NPCs started to appear from neural rosettes, and then NPCs were expanded for 3–4 passages with accutase in neural maintenance medium until day 30, when cells were cryopreserved in the same media with 10% dimethyl sulfoxide (DMSO). Media was supplemented with RevitaCell when splitting the cells till NPCs were obtained.

For astrocyte differentiation, NPCs were cultured in glial expansion medium for 3 weeks to obtain glial precursors. After this time, medium was switched to astrocyte induction media for 3 weeks and, finally, cells were changed to maturation medium for 4 weeks to generate mature astrocytes. Throughout differentiation, cells were passed with accutase when reached confluence and medium was changed partially three times a week. From each cell line, two independent neural differentiations were performed, considering 6 biological replicates for CTRL and 6 for AD samples.

### Establishment of co-cultures between astrocytes and neurons

Astrocytes were seeded at 26,000 cells/cm^2^ in Geltrex-coated plates with astrocyte maturation media. Two days later, NPCs were seeded on top of astrocytes at 26,000 cells/cm^2^ in co-culture medium supplemented with RevitaCell. Co-cultures were maintained in this medium for 25 days, changing the medium partially twice a week.

### Human brain samples

Frontal cortex (lateral prefrontal region) samples were obtained from the Neurological Tissue Bank BioBanco-Hospital clínico-IDIBAPS (Barcelona, Spain). This study was approved by the Portal de Etica de la Investigacion Biomedica de Andalucia (PEIBA) de la Consejeria de Salud from Andalucia (Spain), as well as by the corresponding biobank ethics committees. Subjects were classified according to Braak stage for tau pathology as (1) Braak V-VI (*N* = 6, 79.3 ± 8.4 years, 8.025 h mean postmortem delay) that met the clinical criteria for AD, and (2) Braak II subjects (*N* = 5, 82.6 ± 7.4, 10 h mean postmortem delay) which were age-matched controls without neurological impairments. For the 6 AD patients, one individual was *APOE2/3*, three were *APOE3/3* and two were *APOE3/4*. For the 5 control individuals, one was *APOE2/3*, two were *APOE3/3* and two were *APOE3/4*. Lateral prefrontal cortex samples (from Bregma − 42 mm to −1.3 mm) were fixed in 4% paraformaldehyde for 24–48 h, cryoprotected in sucrose, stored at −80 °C and sectioned at 30 μm thickness on a freezing microtome. Nissl-stained sections showing cytoarchitecture and human brain atlas [21] were used for anatomical delineation.

### Immunofluorescence

Human iPSC-derived astrocytes, previously seeded on Geltrex-coated coverslips in 24-well plates, were washed with PBS and fixed with a 4% paraformaldehyde for 15 min at RT. For fixed human brain samples, for general antigen retrieval, free-floating sections were heated at 80 °C for 20 min in 50mM citrate buffer pH 6.0 before staining.

Human iPSC-derived astrocytes and human brain samples were sequentially incubated in the selected primary antibodies (diluted in PBS with 0,1% triton X-100 and 1% goat or donkey serum, see Supp. Table 8) at 4 °C overnight, and then with the corresponding Alexa fluor 488 or 568-coupled secondary antibodies diluted to 1:500 (or 1:1000 for brain samples, Supp. Table 9) for 90 min at RT in darkness. For nuclear staining, samples were incubated (15 min at RT) with DAPI 1 mg/mL (Sigma-Aldrich, D9542) diluted to 1:1000 (or 1:250 for brain samples) in PBS with 0,1% triton X-100. Finally, cell samples were mounted with ProLong (Invitrogen, P36984) and brain samples sections were embedded in autofluorescence eliminator reagent (Merck Millipore, 2160) and mounted on Superfrost Plus Microscope Slides, coverslipped with 0.01 M PBS containing 50% glycerin and 3% triethylenediamine.

Immunofluorescence images for astrocytic markers, Ki67 and P21, were visualized and acquired with an Olympus VS120 microscope (Olympus VS120 Virtual Slide Microscope) using a 20x objective. Immunofluorescence images for TOMM20 (translocase of outer mitochondrial membrane 20), LC3B (autophagy marker light chain 3B) and synaptic markers were visualized with a Leica Stellaris 8 confocal laser microscope and the TCS NT software (Leica). These images were acquired using a 63x oil immersion objective at a resolution of 1024 × 1024 pixels. To obtain several optical sections on the same Z-axis (Z-stack), images were taken at intervals of approximately 1.2 μm throughout the thickness of the sample. Maximum intensity projection of each Z-stack image was further operated on FIJI software. Images of GFAP (glial fibrillary acidic protein) and H2A.X (H2A histone family member X) of brain sections were also acquired with a Leica Stellaris 8 confocal laser microscope using a 20x or 63x oil immersion objectives at a resolution of 2048 × 2048 pixels. To obtain several optical sections on the same Z-axis (Z-stack), images were taken at intervals of approximately 1 μm throughout the thickness of the sample.

Quantitative Image Analysis. Quantifications of the percentage of positive cells, positive area per cell, soma area and fluorescence intensity per cell were performed using FIJI software. To quantify the area of ​​TOMM20 and LC3B co-localization, the images were analyzed with a semi-automatic macro in FIJI, providing information on the spatial relationship between autophagosome structures and mitochondria. Data were normalized to the number of cells.

Quantifications of H2A.X positive cells (senescent cells), H2A.X positive cells expressing GFAP (senescent astrocytes) and the total cell numbers were performed using a semi-automatic macro in FIJI software. Briefly, a maximum intensity projection was performed and the channels corresponding to DAPI, H2A.X and GFAP markers were separated. DAPI-labeled cell nuclei and H2A.X-positive nuclei were counted using the StarDist plugin [22]. H2A.X-positive nuclei were dilated to 1.89 microns, creating a halo around them. Using mathematical operations, the H2A.X-positive nuclei that had a positive GFAP labeling in the generated halo, corresponding to H2A.X-positive astrocytes, were selected and counted.

### MitoTracker assay

Astrocytes were seeded on Geltrex-coated coverslips in 24-well plates with maturation medium at 40–60% confluency for correct subsequent visualization. MitoTracker Red CMXRos Dye (Invitrogen, M7512) staining was performed 2 days after seeding. Cells were washed with PBS and incubated with medium containing MitoTracker at a concentration of 100 nM for 15 min at 37 °C. Cells were then washed with PBS and fixed with a 4% paraformaldehyde solution for 15 min at RT and washed with PBS. For nuclear staining, astrocytes were incubated with DAPI diluted to 1 µg/mL in PBS 0,1% triton X-100 for 15 min at RT. Finally, samples were mounted with ProLong.

Images acquisition was performed using a Leica Stellaris 8 confocal laser microscope as explained above. Images were obtained from at least 3 randomly assigned regions of interest in each sample. Quantification of mitochondrial content, area and distribution was performed using a semi-automatic macro that processed the images using FIJI software. Briefly, a maximum intensity projection was performed, and the channels corresponding to DAPI and MitoTracker mark were separated. On the latter, the image was improved by enhancing the contrast with a normalization filter. This allowed the limits of each cell to be selected by hand, thus saving 20 independent cell image cutouts from each condition and measuring the area on each of them. In addition, on the MitoTracker mark channel, background correction and contrast enhancement were performed with the CLAHE function, to apply the tubeness function to detect fibrillar structures. Subsequently, this detection was segmented to get a binary image, which was skeletonized with the skeletonize function. With the analyze skeleton function, the information corresponding to each segment was obtained. The parameters of the mitochondrial network of each cell were then obtained, all always limited to the cellular area. Next, the distribution of mitochondria in the cells was analyzed. Using the binary image of the skeleton as a reference, mitochondrial network was analyzed in a halo generated from the edge of the nucleus and proportional to its size. Additionally, the intdent (fluorescence intensity) of the MitoTracker signal for each analyzed cell was obtained.

### BODIPY assay

Astrocytes were seeded on Geltrex-coated coverslips in 24-well plates with maturation medium at 40–60% confluency for correct subsequent visualization. 2 days after seeding, cells were fixed with a 4% paraformaldehyde solution for 15 min at RT and washed with PBS. Astrocytes were incubated in PBS with BODIPY (Thermo Fisher Scientific, D3922) at a concentration of 5 µM for 20 min at RT. For additional stainings, the immunofluorescence procedure was followed. Finally, samples were mounted with ProLong. Images acquisition was performed using a Leica Stellaris 8 confocal laser microscope as explained above.

Lipid droplet staining with BODIPY was also analyzed by flow cytometry. Astrocyte cultures were incubated with maturation media containing BODIPY at a concentration of 5 µM for 20 min at 37 °C. Cells were then washed with PBS, collected with accutase and BODIPY signal was analyzed in a BD FACSVerse flow cytometer (BD Biosciences).

### Phalloidin staining

Phalloidin is a fluorescent conjugate used to label actin filaments in cells. Astrocytes were cultured in 24-well plates with Geltrex-treated coverslips with maturation medium. First, they were fixed with with a 4% paraformaldehyde solution for 15 min at RT, washed with PBS and permeabilized with PBS 0,1% triton X-100 for 1 h at RT. Phalloidin (Sigma-Aldrich, P1951) was added diluted 1:100 in PBS 0,1% triton X-100 and incubated for 2 h at RT. Then, nuclear staining was performed with DAPI at a concentration of 1 µg/mL in PBS 0,1% triton X-100 for 15 min at RT. Finally, samples were mounted with ProLong.

Images were acquired with the Olympus VS120 microscope (Olympus VS120 Virtual Slide Microscope) and analyzed with a semi-automatic macro in FIJI. This procedure provided the parameters related to area, perimeter and area of ​​cytoskeletal fibers of each cell.

### Flow cytometry

Cells were collected using accutase, centrifuged and resuspended in 100 µL FACS (fluorescence-activated cell sorting) buffer (PBS, 0,5% bovine serum albumin (BSA) and 0,02% sodium azide). Then, cells were fixed and permeabilized with Fix & Perm cell permeabilization reagent kit (Life Technologies, GAS003). Primary antibodies were added and incubated for 30 min at 4 °C. Astrocytes were then washed, resuspended in FACS buffer with the secondary antibodies and incubated for 30 min at 4 °C. Finally, after washing with FACS buffer, samples were analyzed in a BD FACSVerse flow cytometer (BD Biosciences).

### Western-Blotting

For proteins detection, cells were collected with accutase, lysed with RIPA buffer and the amount of protein was determined by the BCA (bicinchoninic acid assay) method (Micro BCA Protein Assay, Thermo Fisher Scientific, 23235). Fifteen µg of total protein were diluted in NuPAGE LDS sample buffer (Invitrogen, NP0007), NuPAGE sample reducing agent (Invitrogen, NP0009) and H₂O. Proteins were denatured by heating at 95 °C for 8 min and separated by electrophoresis on a 10% acrylamide Bis-Tris gel (Invitrogen, NP0303BOX). Subsequently, proteins from the gel were transferred to a nitrocellulose membrane (Bio-Rad, 1704159) using a blotting equipment (Trans-Blot Turbo, Bio-Rad). The membrane was washed with TBS-T buffer and blocked with TBS-T 5% BSA for 1 h. After this, the primary antibodies were incubated in TBS-T 5% BSA overnight at 4 °C with shaking. Then, the membrane was washed with TBS-T and secondary antibodies conjugated to the Horseradish peroxidase were incubated in TBS-T for 1 h at RT with shaking and washed with TBS-T. In the case that the secondary antibody was not conjugated to peroxidase, a biotinylated secondary antibody was used and then incubated with a 1:2000 solution of streptavidin (containing peroxidase) (Sigma-Aldrich, E2886) in TBS-T. The molecular mass of the proteins was determined by comparison with a molecular weight standard, PageRuler Plus Prestained Protein Ladder (Thermo Fisher Scientific, 26619). To visualize the labeled proteins, membranes were developed using the SuperSignal West Pico PLUS Chemiluminescent Substrate kit (Thermo Fisher Scientific, 34580) or SuperSignal West Dura Extended Duration Substrate (Thermo Fisher Scientific, 34075) whose reagents contain the peroxidase substrate and will give rise to a signal proportional to the amount of protein in the sample. The signal was measured with the Chemidoc Touch Imaging System (Bio-Rad) using Image Lab software. For quantification, images were processed with the software FIJI. Data were normalized to GAPDH (glyceraldehyde 3 phosphate dehydrogenase) expression.

### Transmission electron microscopy (TEM)

Astrocytes were seeded on Geltrex-coated Thermanox slides in 24-well plates with maturation media at a 70–80% confluence. Two days later, astrocytes were fixed with 2,5% glutaraldehyde in PB buffer for 1 h at RT and postfixed with 2% osmium tetroxide for 1 h at RT. Subsequently, the cell-seeded coverslips were put through a dehydrating series of graded concentrations of ethanol and embedded in Durcupan resine (Sigma-Aldrich, 44610). Semi-thin Sect. (1 μm) were performed and stained with toluidine blue for the identification of regions of interest. Then, ultra-thin Sects. (50–90 nm) were performed and mounted on nickel grids and examined with a JEOL JEM-1400 transmission electron microscope and SightX Viewer software.

### Seahorse

The Seahorse XFe24 extracellular flux analyzer (Agilent Technologies) was used to study the energetic phenotype of astrocytes, which allows real-time analysis of the main energy production pathways of the cell: mitochondrial respiration and glycolysis. These mitochondrial functions were measured by two parameters, oxygen consumption rate (OCR) and extracellular acidification rate (ECAR), respectively. In addition to measuring these parameters under basal conditions, a cellular stress test (Mito Stress Test) was performed, allowing monitoring of OCR by adding modulators of mitochondrial respiration. These modulators were oligomycin (Sigma-Aldrich, O4876), phenylhydrazone (FCCP, Carbonyl cyanide-4-(trifluoromethoxyphenylhydrazone)) (Sigma-Aldrich, C2920), rotenone (Sigma-Aldrich, R8875) and antimycin A (Sigma-Aldrich, A8674).

Astrocytes were seeded on Geltrex-coated Seahorse XFe24 cell plates (Agilent, 100777-004 with maturation medium at a confluence of around 100% 2 days before the experiment. Before measurements, cells were incubated with Seahorse XF DMEM medium (a medium supplemented with glucose, pyruvate and glutamine) (Agilent, 103575-100) at 37 °C for 1 h in the XF Prep Station (Agilent). After this time, medium XF DMEM was changed again, and the equipment was calibrated and prepared with the modulators. Baseline respiration measurements were followed by injection of 1 µM oligomycin, 2µM FCCP, 0,5 µM rotenone and 0,5 µM antimycin. All measurements were normalized to the number of cells and analyzed on Seahorse analytics (Agilent). At the end of the experiment, cells were fixed with a 4% paraformaldehyde solution for 15 min at RT and washed with PBS. For nuclear staining, astrocytes were incubated with DAPI diluted to 1 µg/mL in PBS 0,1% triton X-100 for 15 min at RT. The whole surface of each well was scanned and counted in an Operetta system (PerkinElmer) to determine the number of cells present in each well.

### Mitochondrial superoxide production

To measure mitochondrial superoxide production, flow cytometry was used. Astrocytes were seeded on Geltrex with maturation medium at a confluency around 70–80%. 4 days after seeding, cells were washed with HBSS (Gibco, Thermo Fisher Scientific, 14025-050) and they were incubated with Mitosox Red superoxide mitochondrial indicator (Invitrogen, M36007) at a concentration of 5 µM in HBSS for 30 min at 37 °C. After this time, cells were washed with HBSS, collected with accutase and resuspended in HBSS to perform flow cytometry.

### Astrocyte activation

To promote astrocytic activation, astrocyte maturation media was supplemented with 400 ng/mL of C1q (complement component 1q) (Sigma-Merck: 204876), 3 ng/mL of IL (interleukin) 1a (Peprotech 200–01 A) and 30 ng/mL of TNFa (tumor necrosis factor alpha) (Peprotech 300–01 A) (TIC cocktail) and added to the cells for 24 h previous to analysis.

### Glutamate uptake

Astrocytes were cultured at 70–80% confluence in geltrex-coated 24-well plates with maturation media. The assay was performed 3 days after seeding. Cells were incubated with HBSS at 37 °C for 10 min and then glutamate was added at a concentration of 100 µM in HBSS buffer for 1 h at 37 °C. The absorption was stopped by removing the solution with glutamate. This solution was analyzed with the MAK004 glutamate assay kit (Sigma-Aldrich) to get the glutamate concentration measuring absorbance at 450 nm. The glutamate concentration of each solution of each cell line was subtracted from the concentration value of a control well that does not contain cells and whose consumption was none. In addition, the data were normalized according to the number of cells in each well, thus obtaining the absorption data per cell. To do this, after the experiment, cells were fixed with a 4% paraformaldehyde solution for 15 min at RT and washed with PBS. For nuclear staining, astrocytes were incubated with DAPI diluted to 1 µg/mL in PBS 0,1% triton X-100 for 15 min at RT. The plates were scanned and counted in an Operetta system.

### RNA extraction, cDNA synthesis and RT-qPCR

Total RNA was purified using the NZY Total RNA Isolation kit (NZYtech, MB13402) following manufacturer instructions. After concentration and integrity validation (NanoDrop 1000, Thermo Fisher Scientific, MA USA), cDNA was generated using 0.5–1.5 µg of RNA with High Capacity cDNA Reverse Transcription Kit (Applied Biosystems, 4368814) and qRT-PCR was performed in technical triplicates in a Lightcycler 96 (Roche) employing the QUANTIMIX HotSplit Easy kit (Biotools) and primers mix at final concentration of 250 nM. Gene expression (cycle threshold) values were normalized based on the GAPDH housekeeping gene and for the quantification of cDNA level, the comparative double delta cycle threshold (Ct) method (2 − ΔΔCt) was used. See Supp. Table 12 for a list of all RT-qPCR primers used in this study. The gene set score was used to quantitatively calculate variations in expression of different markers, associated with the same cell type [23]. Concisely, the gene set score was calculated for each sample ‘j’ using the value ‘eij’, which is the normalized expression level of gene ‘i’ in sample ‘j’. We calculate the center gene expression matrix “cij” with the following equation: $\:cij=eij-1ns\sum\:jeij$; where “ns” is the number of samples. The gene set score for sample “j” (Sj) is defined as the average of the matrix $\:Sj=1ng\sum\:icij$; where “ng” is the number of genes which constitutes the cluster.

### Transcriptome studies and analyses

Live cells for mRNA-seq analysis were frozen in N2B27 medium supplemented with 10% dimethyl sulfoxide. A resuspension buffer containing 10 mM Tris-HCl, 10 mM NaCl, 3 mM MgCl2, 0.1% Tween-20, and 1x RNasin Plus (Promega, cat# N2611) was added to frozen cell suspensions, after which they were allowed to thaw on ice. Live and dead cells were counted with trypan blue staining, and ≥ 100,000 total cells were aliquoted to microcentrifuge tubes for RNA extraction. Samples were pelleted by centrifugation (500 x g for 5 min), supernatant was removed, and pellets were resuspended in TRI reagent (Zymo Research, cat# R2050-1–50). Resuspensions were flash frozen in a slurry of ethanol and dry ice and stored at −80 °C until extraction. RNA was extracted from pellets using the RNeasy Mini Kit (Qiagen cat# 74106), and high (> 7) RIN scores were verified using Agilent RNA ScreenTape (Agilent Technologies, cat# 5067–5578) on the Agilent Tapestation. Sequencing libraries were prepared with the Illumina Stranded mRNA Prep, Ligation Kit (Illumina, cat# 20040534) and sequenced using an Illumina NovaSeq 6000 machine to a minimum of 6 gigabases (Gb) depth per sample with 150 + 150 cycles paired-end reads.

The quality of the paired-end reads from *APOE4/4* (AD) and *APOE3/3* (CTRL) patient-derived astrocytes was analyzed by FastQC (v0.11.9) (https://www.bioinformatics.babraham.ac.uk/projects/fastqc/), and the adaptors sequence were trimmed with Cutadapt (v3.5) [24]. The trimmed reads were mapped with HISAT2 (v2.2.1) [25] to the reference genome (transcripts from human genome GRCh38 Release 115). The resulting BAM files were converted to SAM format and indexed and, then, used for read counting on FeatureCounts (Subread package v2.03). DESeq2 package (v1.46) [26] on R Studio (v2024.12.1 + 563) was used to discard lowly expressed genes (less than 10 counts in each sample), apply quality control [24], perform differential expression (DE) analysis comparing AD vs. CTRL samples for each gene, and generate a normalized count matrix. The group comparison produced a matrix with the output of the statistical test and was used to generate a rank list of genes sorted by the test statistic (test value). To identify differences in biological processes between AD and CTRL samples, gene set enrichment analysis (GSEA; v4.2.3) [27] was applied on the rank list from above. The following molecular signatures were analyzed: Hallmarks Gene Sets (manually adding the senescence profiles) and KEGG-Legacy subset. The top ranked genes from the pathways of interest were used to produce heatmaps representations, based on the values of the normalized count matrix.

### Statistical analysis

The distribution of the data was evaluated with the Shapiro-Wilk test. If the distribution was normal, parametric tests were used. To compare two independent groups, a t-test was performed. To compare more than two independent groups, the ANOVA test (analysis of variance) was performed, followed by Tukey’s multiple comparison test, to determine which groups were significantly different.

For data that did not follow a normal distribution, non-parametric tests were used. To compare two independent groups, the Mann-Whitney test was used. For comparisons of more than two independent groups, the Kruskal-Wallis test, followed by Dunn’s multiple comparison test, were performed to determine which groups were significantly different.

For analysis of paired samples (treatment and non-treatment), which did not follow a normal distribution, the Wilcoxon test was performed.

Results were plotted and analyzed using GraphPad Prism 9.4.1 software. Data are shown as bar graphs, reflecting the individual mean values ​​of each sample and the standard deviation (SD). Regarding the level of significance, it has been considered that the differences are statistically significant when a *p* < 0.05 value is obtained, and the level of significance has been shown as follows: *p < 0.05, **p < 0.01, ***p < 0.001 and ****p < 0.0001.

## Results

### Generation and phenotypic characterization of hiPSC-derived AD astrocytes

**Fig. 1:**
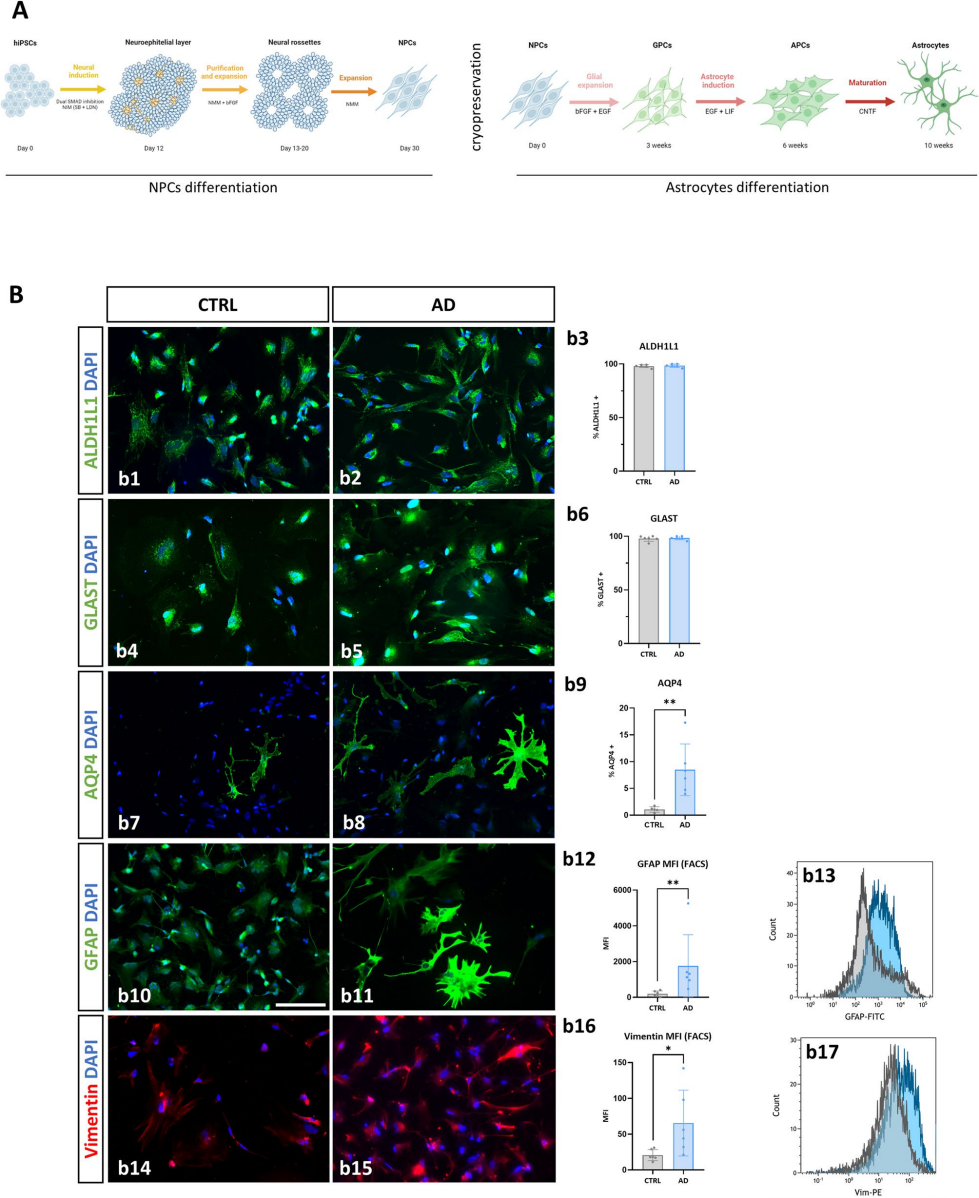
Generation and characterization of hiPSCs-derived astrocytes from AD patients (*APOE4/4*) and control (CTRL) individuals (*APOE3/3*). **A** Layout of the protocol for the generation of astrocytes from human iPSCs. **B** Representative immunofluorescence images and quantification in both groups of astrocytes of the astroglial markers ALDH1L1(b1-3), GLAST (b4-6) and AQP4 (b7-9). Representative immunofluorescence images, quantification of the median intensity fluorescence (MFI) by flow-activated cell sorting (FACS) and comparative FACS histograms of two representative astrocyte lines analyzed (in gray a CTRL line and in blue an AD line) for the markers GFAP (b10-13) and Vimentin (b14-17). Nuclei were counterstained with DAPI (blue). Scale bar: 100 μm. Individual values ​​for each cell line differentiation are represented, along with the mean and standard deviation. *p < 0.05, **p < 0.01

As serum produces enormous changes on astrocytes at both phenotypic and functional levels [28], we adapted a published serum-free protocol [20] for the generation of hiPSC-derived astrocytes (Fig. 1A). Following this method, we were able to generate a pure population of astrocytes from hiPSC lines from *APOE4/4* AD patients (AD astrocytes from now on) and from age-matched *APOE3/3* cognitively healthy subjects (CTRL astrocytes from now on), which expressed canonical astrocytic markers as ALDH1L1 (aldehyde dehydrogenase 1 family member L1) (Fig. 1B b1-3) and GLAST (Fig. 1B b4-6). We also evaluated the expression of APOE between the cell extracts of both groups finding no differences (Supp. Fig. 1).

Next, we evaluated the expression of astrocyte reactive markers up-regulated in AD brains [29], such as AQP4 (aquaporin 4) (Fig. 1B b7-9), GFAP (Fig. 1B b10–13) and Vimentin (Fig. 1B b14–17). The percentage of AQP4-positive AD astrocytes was significantly higher (8.5 ± 4.8%) in comparison with those derived from controls, where the expression of this marker was almost absent (1.1 ± 0.5%, *p* < 0.01). In addition, we found that, although expressed by most of the cells, the fluorescence intensity (median fluorescence intensity, MFI, assessed by FACS) of the expression of the reactive markers GFAP (Fig. 1B b12–13) and Vimentin (Fig. 1B b16–17), was significantly increased (8.8 and 3.2 fold, respectively) in AD astrocytes with respect to CTRL astrocytes (1757 ± 1746 vs. 199.2 ± 158.1 for GFAP, *p* < 0.01; and 65.5 ± 45.9 vs. 20.7 ± 7.7 for Vimentin, *p* < 0.05). These results suggest that AD astrocytes in basal conditions show an increased expression of in vivo astroglial AD-associated reactive markers. 

To verify AD astrocytes present a reactive phenotype, we decided to evaluate at the transcriptome level the transforming growth factor beta (TGFb) signaling, as it is crucial for astrocytes because it regulates their development, reactivity, function, and neuroprotective roles within the CNS [30]. We found that this pathway was enriched between AD astrocytes (with a Normalized Enrichment Score (NES) of 1.99, Supp. Fig. 2). These results indicate that AD astrocytes in basal conditions show a reactive state in comparison with CTRL-derived astrocytes.

### Mitochondrial network is altered in hiPSC-derived AD astrocytes

**Fig. 2:**
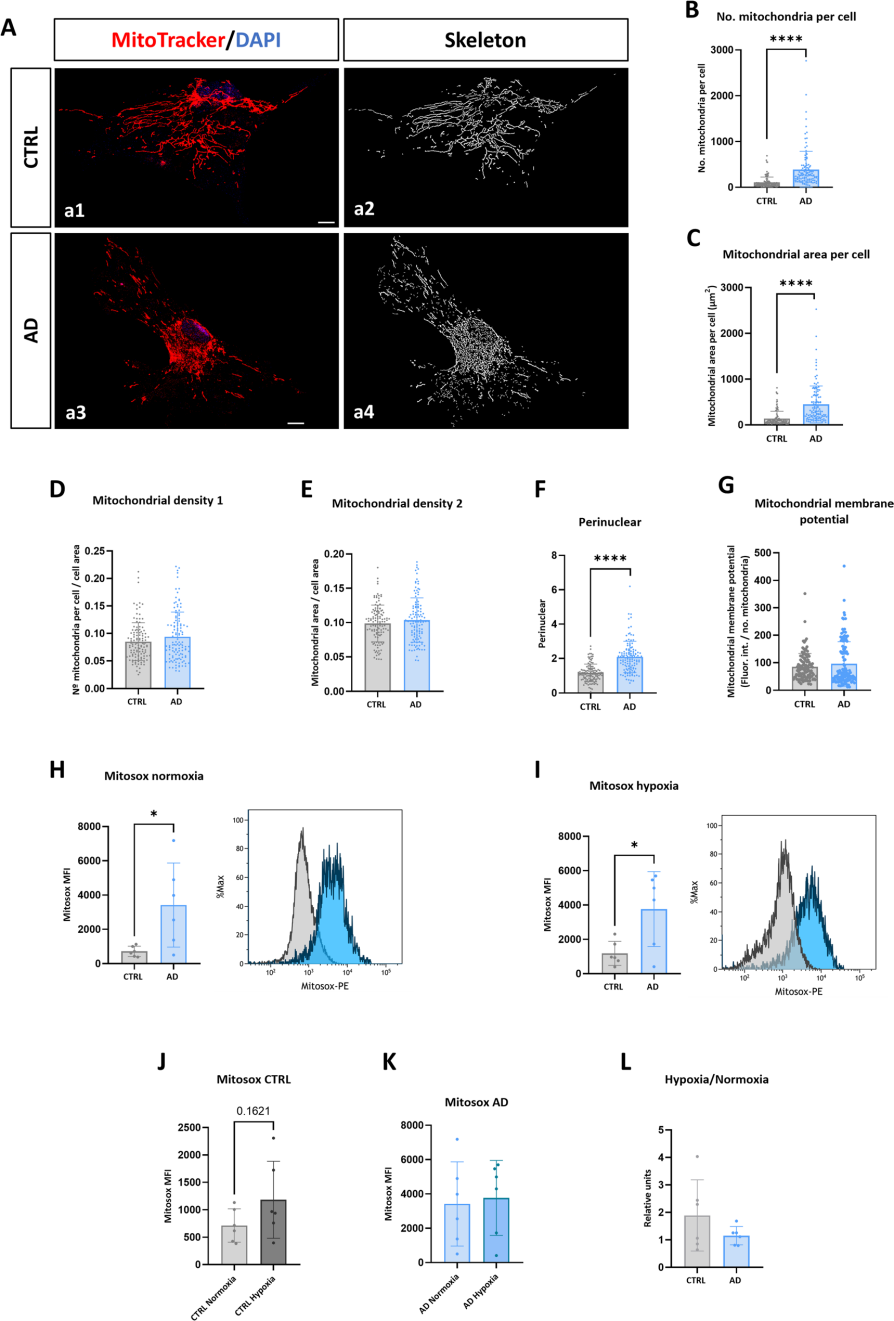
Analysis of the mitochondrial network and mitochondrial ROS production of hiPSC-derived AD astrocytes. **A** Immunofluorescence analysis by confocal microscopy of the mitochondrial marker MitoTracker in CTRL (a1) and AD (a3) astrocytes. On the right, the skeletonization (a2 and a4) of the mark is represented for better visualization and quantification. Nuclei were stained in blue with DAPI. Scale bar 10 μm. **B** Quantification of the number of mitochondria per cell, (**C**) area of ​​the mitochondrial network per cell, (**D**) mitochondrial density 1 referred to cell area and (**E**) mitochondrial density 2 in relation to the cell area. **F** Quantification of the distribution of mitochondria through a ratio between the percentage of mitochondrial area around the nucleus with respect to the percentage that this area represents with respect to the cellular area (perinuclear location of mitochondria). **G** Quantification of mitochondrial membrane potential through the fluorescence intensity of the MitoTracker signal in CTRL and AD astrocytes. For panels B to G, *N* = 20 individual cells were analyzed per cell line differentiation, along with mean and standard deviation. **H** FACS quantification of the MFI (median fluorescence intensity) of the Mitosox signal in the two study groups under normoxia conditions and comparative histogram of two representative astrocyte lines analyzed, in grey a CTRL line and in blue an AD line. **I** FACS quantification of the MFI of Mitosox in the two experimental groups under hypoxia conditions and histogram, in grey a CTRL line and in blue an AD line. **J** Quantification of the MFI of Mitosox in CTRL astrocytes comparing normoxia and hypoxia conditions. **K** Quantification of Mitosox MFI in AD astrocytes comparing normoxia and hypoxia conditions. **L** Analysis of the hypoxia/normoxia ratio. Individual values ​​for each cell line differentiation are shown, along with mean and standard deviation. *p < 0.05. ****p < 0.0001

Astrocytes have a key role in maintaining and regulating brain energy metabolism [31] which is highly compromised in AD. Since astroglial metabolic dysfunction have been reported in AD brains [32], we next focused on addressing potential alterations in the mitochondrial network in AD astrocytes. First, we assessed mitochondrial network by MitoTracker staining (Fig. 2A) and evaluation by confocal microscopy and skeletonization of the signal. As can be seen in Fig. 2 (B and C), more than a three-fold increase in the number and the area of mitochondria per cell was detected in AD astrocytes in comparison with CTRL astrocytes (382.9 ± 403.5 vs. 105.1 ± 117.6, *p* < 0.0001 for mitochondria number and 447.3 ± 404.5 vs. 138.3 ± 160.2, *p* < 0.0001 for mitochondria area). However, when we normalized the number and the area of mitochondria with respect to cell size, the density of mitochondria per cell did not vary between the two study groups (Fig. 2D and E). Moreover, a significant change in the spatial distribution of mitochondria within the cell was observed. Meanwhile in CTRL astrocytes these organelles were distributed homogeneously within the cytoplasm, in the case of AD astrocytes, they exhibited a preferential clustering around the perinuclear area. To be able to measure this, we defined the perinuclear area (as the nuclei plus 20% of its area) and assessed the percentage of the total mitochondria that located within this area (Fig. 2F). Interestingly, a significant increased perinuclear distribution of mitochondria was found in AD astrocytes in comparison to CTRL astrocytes (2.1 ± 0.9 vs. 1.1 ± 0.5, *p* < 0.0001). These data indicate that in AD astrocytes mitochondria are abnormally distributed within the cell cytoplasm, concentrating around the perinuclear area.

### hiPSC-derived AD astrocytes produce high levels of mitochondrial reactive oxygen species (ROS)

Next, we assessed the mitochondrial membrane potential produced by measuring the intensity of the MitoTracker signal. No differences in the membrane potential at the individual mitochondrion level were found between AD and CTRL astrocytes (Fig. 2G).

Production of reactive oxygen species (ROS) by mitochondria is consequence of mitochondrial malfunction and has important consequences for cellular homeostasis and response to stress [33]. Using the intensity of the Mitosox signal, we measured the mitochondrial ROS production in both groups of astrocytes (Fig. 2H). Culturing the cells in normoxic conditions (21% O_2_), we observed a significant 4.8-fold increase in the Mitosox signal within AD astrocytes in comparison with control ones (3415 ± 2452 MFI for AD vs. 713.8 ± 303.7 MFI for controls, *p* < 0.05), indicating that under these basal conditions ROS production in AD astrocytes is exacerbated.

Within the brain, there are different areas which suffer from local and temporal oxygen reduction, which has been called hypoxic pockets [34], a fact supposed to be more prevalent in the AD brains since hypoxia is a significant risk factor for the disease [35]. To assess whether hypoxia compromises mitochondrial function in AD astrocytes, we cultured astrocytes in hypoxic conditions (1% O_2_) for 3 days and evaluated ROS production afterwards. Under hypoxia, AD astrocytes displayed higher Mitosox signal in comparison with control astrocytes (Fig. 2I). As expected, hypoxia increased ROS production in CTRL astrocytes (65% of increase, from 713.8 ± 303.7 to 1182 ± 701.6 MFI), but almost without modifying these values in AD astrocytes (10% of increase, from 3415 ± 2452 to 3765 ± 2184 MFI) (Fig. 2J and K). In fact, the hypoxia/normoxia Mitosox signal ratio was about 2 (1.89) for CTRL astrocytes and close to 1 (1.15) for AD astrocytes (Fig. 2L), suggesting that AD astrocytes had no more capacity of modifying ROS production under hypoxic stress.

### Mitochondrial dynamics is skewed towards mitochondrial fusion in hiPSC-derived AD astrocytes

**Fig. 3:**
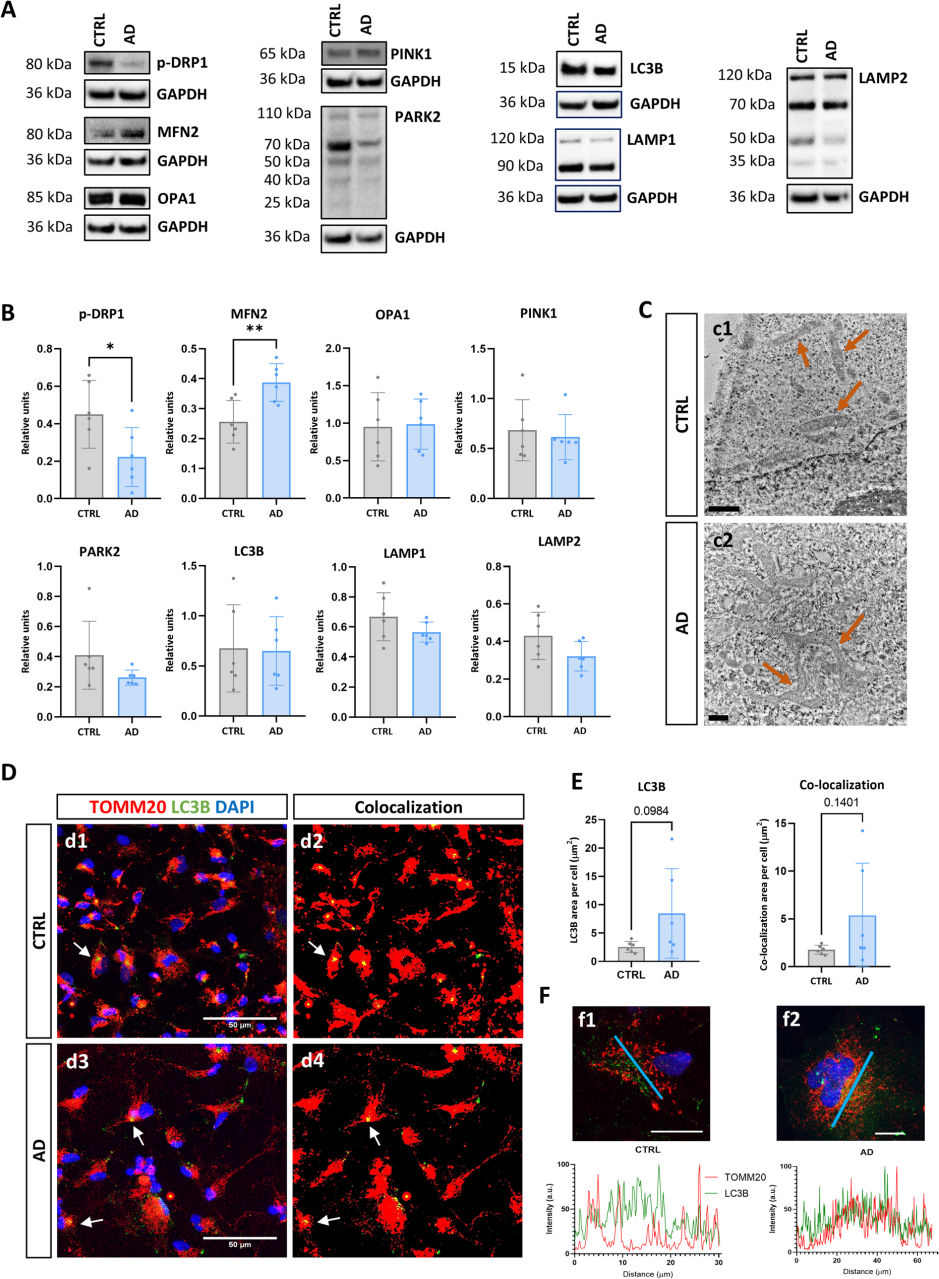
Analysis of mitochondrial fusion, fission and mitophagy processes in hiPSC-derived AD astrocytes. **A** Western blots of the fission marker p-DRP1, fusion markers MFN2 and OPA1 and the markers involved in mitophagy PINK1, PARK2, LC3B, LAMP1 and LAMP2. **B** Quantification of the expression of these proteins relative to GAPDH. Individual values ​​for each cell line differentiation are shown, along with mean and standard deviation. **C** Representative images of transmission electron microscopy showing mitochondria (arrows) from CTRL (c1) and AD (c2) astrocytes. Scale bars 1 and 0.5 μm, respectively. **D** Representative confocal microscopy images of double immunofluorescence for the mitochondrial marker TOMM20 (red) and the autophagosome marker LC3B (green), as well as the co-localization mark of both after image processing (yellow), in CTRL (d1 and d2) and AD (d3 and d4) astrocytes. Areas with high co-localization of both markers are indicated with arrows. Nuclei stained blue with DAPI. Scale bar: 50 μm. **E** Quantification of LC3B expression and the area of ​​co-localization of TOMM20 and LC3B in the two study groups. Individual values ​​for each cell line differentiation are shown, along with mean and standard deviation. **F** Representative confocal microscopy of double immunofluorescence for TOMM20 (red) and LC3B (green) in a CTRL (f1) and AD (f2) astrocytes, as well as a histogram depicting the fluorescence intensity of both markers throughout the cell cytoplasm (blue line shown in the image). Scale bar: 20 μm. Nuclei stained blue with DAPI. **p < 0.01

Mitochondria are highly dynamic organelles which are continuously suffering fusion and fission processes to respond to local energy demands and signals [36]. An alteration in these fusion/fission processes may be a reflection of mitochondrial malfunction. To assess this, we evaluated by western blot (WB) a series of key proteins involved in this mitochondrial dynamic (Fig. 3A and B). The expression of Ser616-phosphorylated Drp1 (dynamin-related protein 1) protein (involved in mitochondrial fission) was significantly diminished in AD astrocytes (50% reduction, *p* < 0.05) but, on the contrary, the expression of the Mitofusin 2 (MFN2, involved in mitochondrial fusion) protein was increased in this group of astrocytes (50% of increase, *p* < 0.05). Notably, the expression of the inner mitochondrial membrane (IMM) protein (OPA1 optic atrophy gene 1), also involved in mitochondrial fusion, did not change between the groups, suggesting that in AD astrocytes the mitochondrial dynamics was skewed towards the promotion of mitochondrial fusion, but this process may be incomplete. In fact, employing transmission electron microscopy (TEM), we found the presence of long-fused mitochondria only in AD astrocytes (Fig. 3C), corroborating this phenomenon.

In addition to fusion and fission processes, mitochondria biogenesis and degradation are two pathways continuously occurring in active cells. In damaged mitochondria (those more prone to be degraded), PINK1 (PTEN-induced kinase 1) accumulates in the outer mitochondrial membrane (OMM) and leads to the accumulation of Parkin (PARK2), which ubiquitinate other substrates, leading to the binding of LC3B^+^ phagosome which, after fusing with LAMP1 (lysosomal associated membrane protein 1) and LAMP2 (lysosomal associated membrane protein 2) positive lysosomes, leads to the degradation of damaged mitochondria [37]. Unexpectedly, despite presenting higher levels of mitochondrial ROS (Fig. 2H), no upregulation of proteins conducting to mitochondrial degradation was detected in AD astrocytes (Fig. 3A and B).

Nonetheless, we wondered whether, without modifying the expression level of the proteins involved, an increased number of mitophagic events were occurring in AD astrocytes. For that, we co-immunostained mitochondria (TOMM20 marker) with the LC3B autophagosome protein and assessed LC3B expression and co-expression of both markers in both types of astrocytes. As can be observed in Fig. 3D and E, we quantified a higher expression (although not statistically significant) of LC3B and its co-localization with TOMM20^+^ mitochondria in AD astrocytes, showing both markers a significant spatial overlay in the case of AD astrocytes (Fig. 3F). Together, these data propose that in AD astrocytes, mitochondrial dynamics is skewed towards mitochondrial fusion and there is a clear tendency suggesting that disease astrocytes are trying to degrade these altered mitochondria.

### hiPSC-derived AD astrocytes present higher metabolic rates and capacities than control astrocytes

**Fig. 4:**
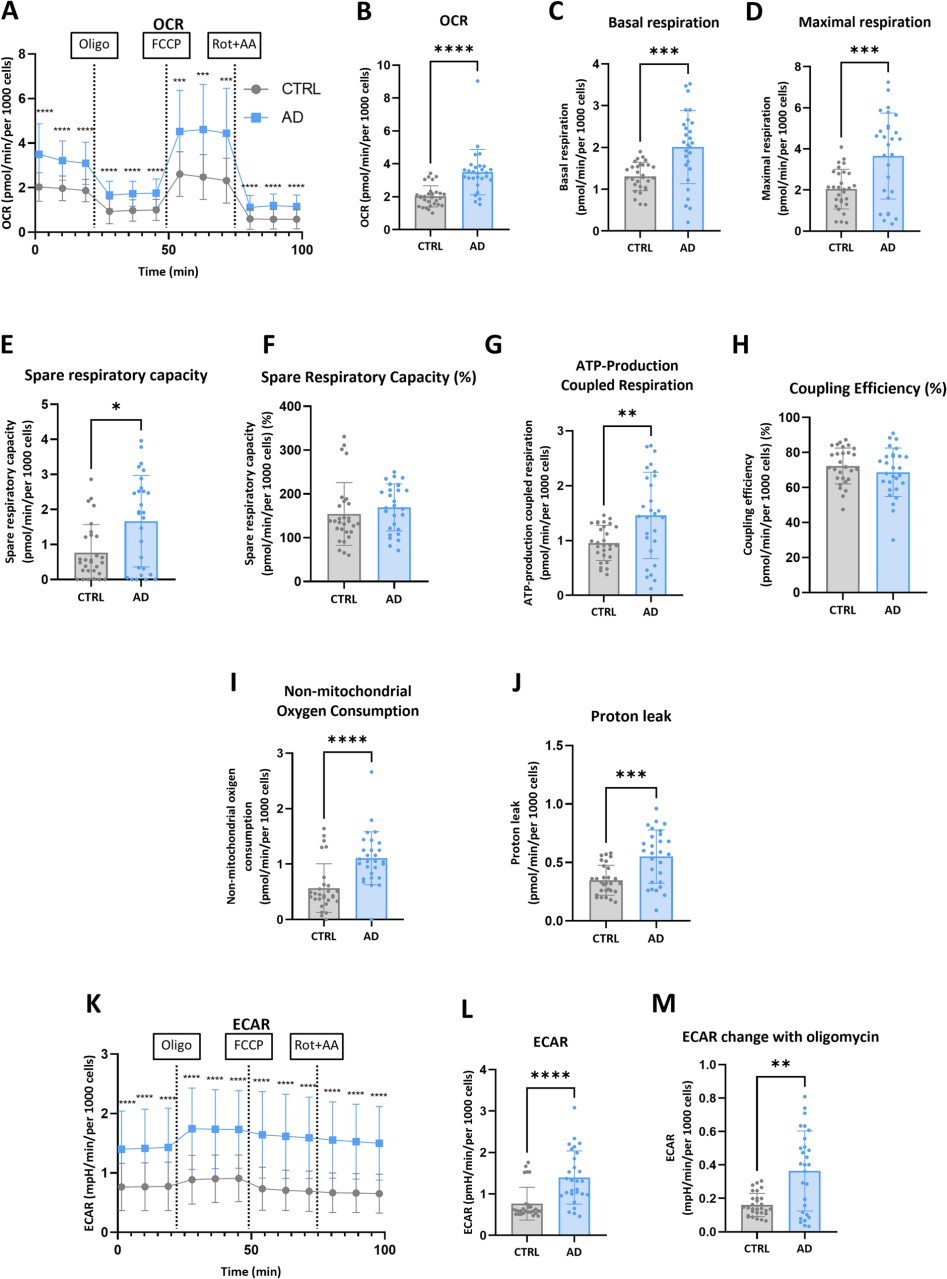
Analysis of the metabolic profile of hiPSC-derived AD astrocytes using Seahorse. (**A**) Study of mitochondrial respiration by measuring the oxygen consumption rate (OCR) over time and (**B**) its quantification, as well as the mitochondrial respiration parameters measured in the Mito Stress test: (**C**) basal respiration, (**D**) maximal respiration, (**E**) respiratory reserve capacity, (**F**) respiratory reserve capacity (%), (**G**) respiration coupled to ATP production, (**H**) coupling efficiency, (**I**) non-mitochondrial oxygen consumption and (**J**) proton leak. (**K**) Study of glycolysis by measuring the extracellular acidification rate (ECAR) over time and (**L**) its quantification. (**M**) Analysis of the increase in ECAR after the addition of oligomycin. The values ​​corresponding to five replicates of each cell line differentiation are represented, together with the mean and standard deviation. *p < 0.05, **p < 0.01, ***p < 0.001, ****p < 0.0001

Since mitochondria play a fundamental role in cellular energy metabolism, mitochondrial metabolic function was evaluated. To assess metabolism, we employed the Mito Stress Test using the seahorse technology, which blocks mitochondrial electron transport chain at different points to assess several parameters associated with mitochondrial functioning at basal and stress conditions (Fig. 4). Surprisingly, at basal conditions, we observed a higher oxygen consumption rate (OCR) in AD astrocytes (Fig. 4A-C), indicating a higher basal metabolic level for these astrocytes. Notably, when the FCCP compound was applied (an uncoupling agent that disrupts the mitochondrial membrane potential, leading to the mitochondrial transport chain to work at its maximum), much higher OCR levels were obtained for AD astrocytes (Fig. 4D), implying also that these cells were able to produce a higher response when needed, a parameter directly influencing the spare respiratory capacity (Fig. 4E and F). Notably, these increases in OCRs were coupled with ATP (adenosine triphosphate) production (Fig. 4G), but with similar coupling efficiency between the two groups of astrocytes (Fig. 4H). These higher values for AD astrocytes were also true for non-mitochondrial oxygen consumption and proton leak (Fig. 4I and J), indicating that the higher oxygen consumption in AD astrocytes was not totally due to mitochondrial energy production.

On the other hand, AD astrocytes presented higher extracellular acidification rates in all assayed conditions (ECAR, Fig. 4K and L). Notably, when mitochondrial electron transport was blocked with oligomycin, higher ECAR increase was observed within AD astrocytes, suggesting also that these cells have a higher capacity of increasing their glycolytic activity in response to oxidative phosphorylation malfunctioning (Fig. 4M). Together, the seahorse data indicate that AD astrocytes present higher metabolic rates in all conditions tested and that these higher metabolic rates were not always coupled with mitochondrial ATP production.

### hiPSC-derived AD astrocytes present a senescent phenotype

**Fig. 5:**
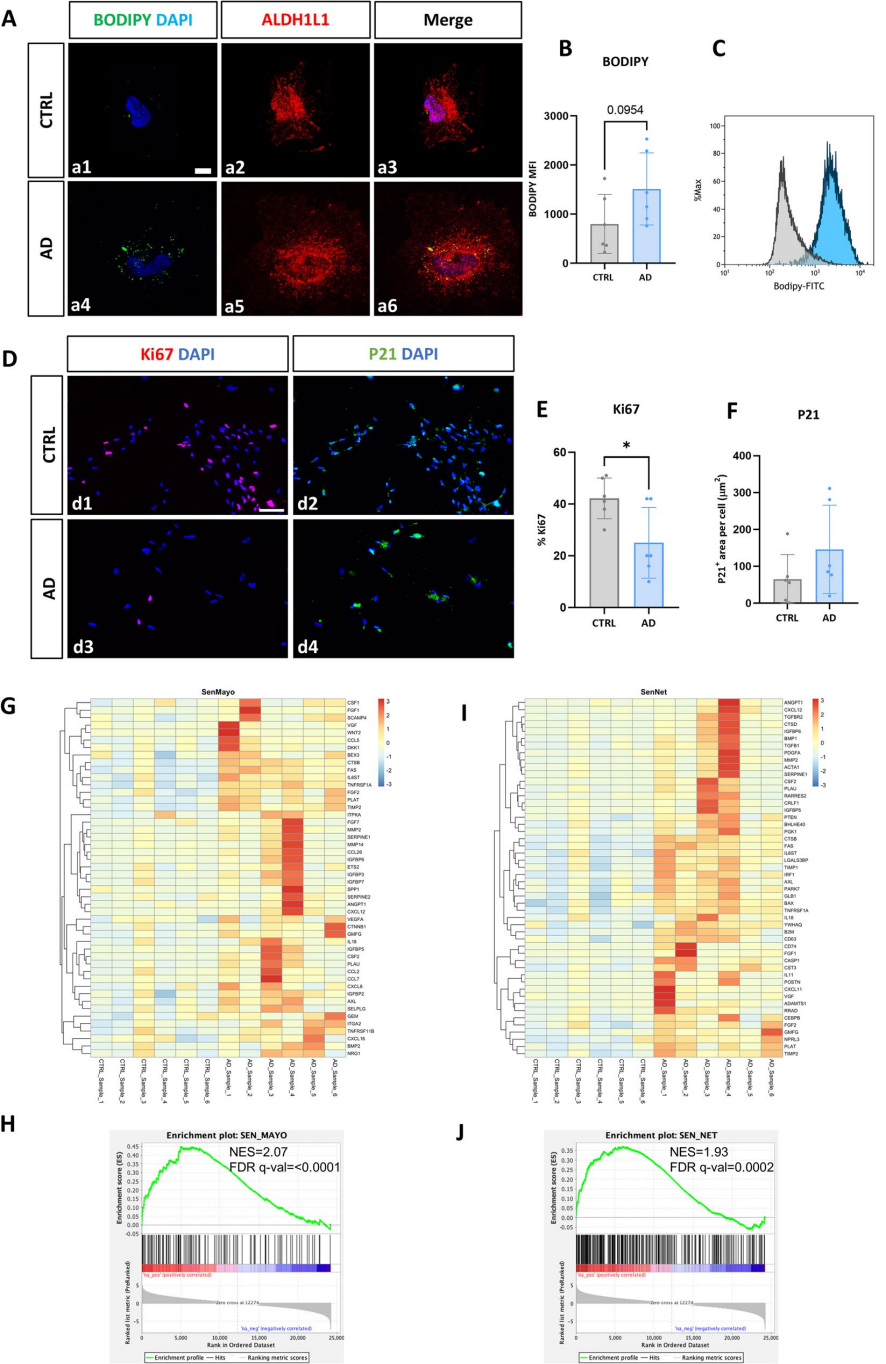
Analysis of the senescent phenotype of hiPSC-derived AD astrocytes. (**A**) Representative immunofluorescence images obtained by confocal microscopy of the lipid droplet marker BODIPY in CTRL (a1-a3) and AD (a4-a6) astrocytes. Nuclei stained blue with DAPI. Scale bar: 10 μm. (**B**) FACS quantification of the median fluorescence intensity (MFI) of BODIPY in CTRL and AD astrocytes, and (**C**) comparative histogram of two representative astrocyte lines, a CTRL line in grey and an AD line in blue. Individual values ​​for each cell line differentiation are represented, together with the mean and standard deviation. (**D**) Representative immunofluorescence images of the expression of the proliferation marker Ki67 and the senescence marker P21 in CTRL (d1 and d2) and AD (d3 and d4) astrocytes. Nuclei stained with DAPI. Scale bar: 50 μm. (**E**) Immunofluorescence quantification of the expression of Ki67 in CTRL and AD astrocytes. (**F**) Immunofluorescence quantification of the P21 expression in CTRL and AD astrocytes. Individual values ​​for each cell line differentiation are represented, along with the mean and standard deviation. **G**-**J**) Gene set enrichment analysis (GSEA) of recently defined genes associated with cell senescence described in [42] (SenMayo) and [39] (SenNet). Heatmaps (**G** & **I**) and enrichment plots (**H** & **J**) of both set of genes with the 48 and top 50 ranking leading edge genes, respectively. NES: Normalized Enrichment Score. *p < 0.05

Mitochondrial dysfunction is closely related to cell senescence. As heightened energy-producing oxidative metabolism, decreased mitochondrial fission, altered mitophagy and higher production of mitochondrial ROS are features of senescent cells [38, 39], we wanted to evaluate whether AD astrocytes may present a senescent phenotype which may explain these mitochondrial alterations. It has been reported that senescent astrocytes accumulate different substances such as lipid droplets [40]. To assess that, we performed BODIPY staining in generated astrocytes and observed a notable accumulation of lipid droplets only in AD astrocytes (Fig. 5A-C). Next, as senescent cells are characterized by a reduced capacity of proliferation and the emergence of markers involved in cell cycle arrest as p21 [41], we evaluated by immunofluorescence the presence of Ki67+ proliferating cells (Fig. 5D d1 and d3) and p21+ cells (Fig. 5D d2 and d4) in both groups of astrocytes (AD and controls). Notably, we found a significant 60% reduction in the number of Ki67+ cells (Fig. 5E) and a 2.3-fold increase in the expression of the p21 marker within the AD astrocytes (Fig. 5F).

On the other hand, we also assessed cell size as cell hypertrophy is another feature of senescent cells [41]. In fact, we observed a 3.2-fold increase in cell area (*p* < 0.0001) and 2.3-fold increase in cell perimeter (*p* < 0.0001) in AD astrocytes, characterized also by an augmentation in the number of actin fibers in their cytoskeleton (2.8-fold increase, *p* < 0.0001; Supp. Fig. 3), suggesting that AD astrocytes are hypertrophic in comparison with those astrocytes derived from cognitively healthy controls.

To confirm these observations, we performed RNAseq and gene set enrichment analysis (GSEA) of recently reported markers associated with cell senescence [39, 42]. As can be observed in Fig. 5G-J, for both sets of genes, we found a notable and significant increase of senescent genes within AD astrocytes in comparison with astrocytes derived from healthy donors (NES values of 2.07 and 1.93, respectively). We validated the expression of the most influential genes in the acquisition of a senescent phenotype [42] and their gene score by qPCR (Supp. Fig. 4), validating the RNAseq data. Together, these data suggest that AD astrocytes, in basal conditions without any stimulus, present a senescent phenotype.

### hiPSC-derived AD astrocytes in basal conditions show a reactive and pro-inflammatory profile

**Fig. 6:**
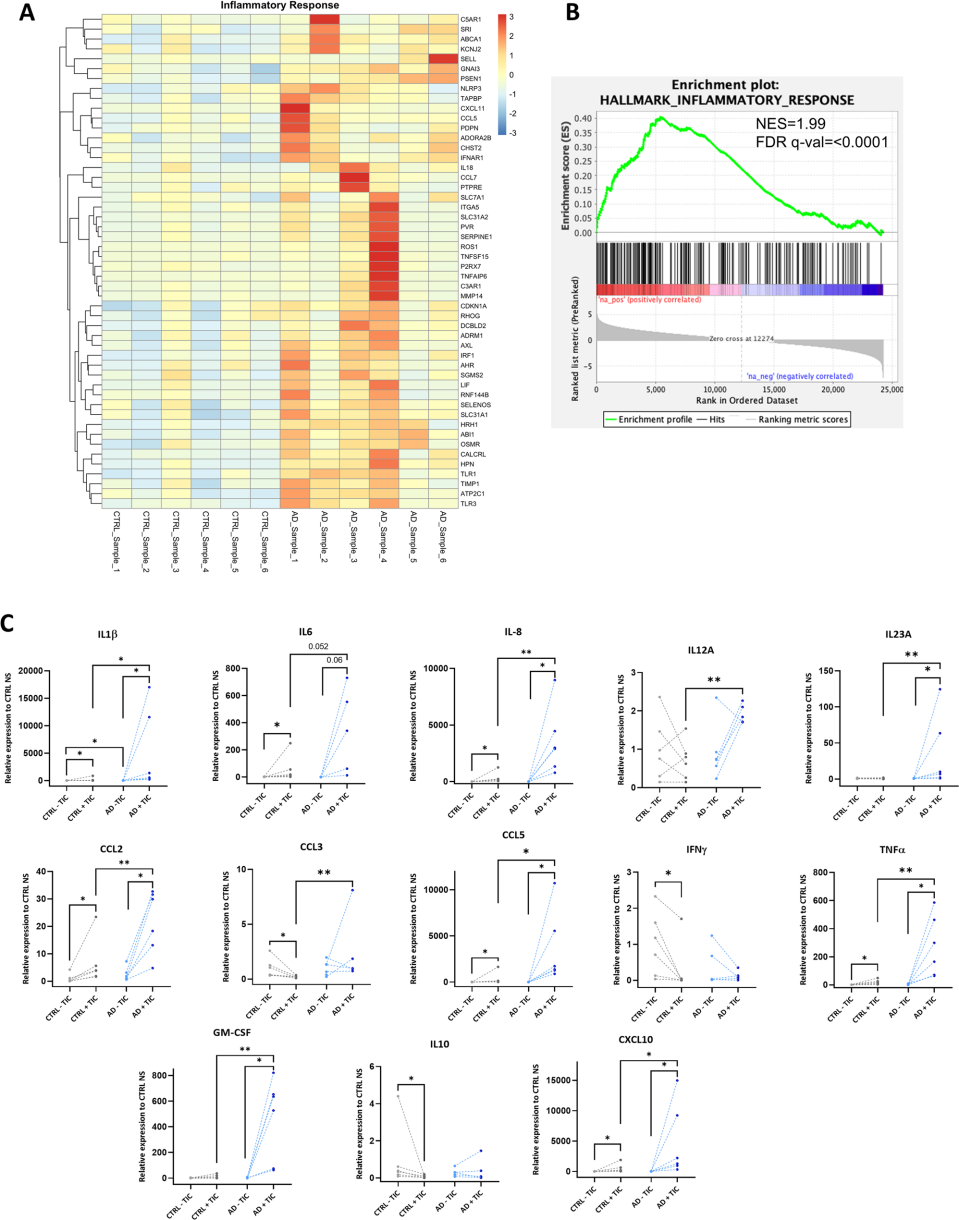
Expression of inflammatory cytokines and chemokines by human iPSC-derived AD astrocytes. **A**-**B** Gene set enrichment analysis (GSEA) of the inflammatory response showing the heatmap (**A**) and the enrichment plot (**B**) of the top 50 ranking leading edge genes. NES: Normalized Enrichment Score. **C** Evaluation by qPCR of the gene expression of pro- and anti-inflammatory cytokines and chemokines after inflammatory stimulation (addition of TNFα, IL1α and C1q for 24 h) of astrocytes. IL1β (interleukin 1β), IL6 (interleukin 6), IL8 (interleukin 8), IL12A (interleukin 12 A), IL23A (interleukin 23 A), CCL2 (C-C motif chemokine ligand 2), CCL3 (C-C motif chemokine ligand 3), CCL5 (C-C motif chemokine ligand 5), IFNγ (interferon γ), TNFα (tumor necrosis factor α), GM-CSF (granulocyte macrophage colony-stimulating factor), IL10 (interleukin 10), CXCL10 (CXC motif chemokine ligand 10). Individual values ​​for each cell line differentiation are shown. *p < 0.05, **p < 0.01

One of the most striking features of senescent cells is that they display an altered expression of cytokines and chemokines, what is called the senescence-associated secretory phenotype (SASP), which compromise tissue homeostasis [39]. To assess differences in the cytokine and growth factors production between the two groups of astrocytes, we performed GSEA to evaluate the expression of the inflammatory response. As can be observed in Fig. 6A and B, an enrichment of inflammatory genes was upregulated (with a NES of 1.99) in AD astrocytes compared to controls. In addition, we evaluated several pro- and anti-inflammatory cytokines, chemokines and growth factors expression by qPCR in basal conditions. As can be seen in Supp. Fig. 5, we found a higher expression of these cytokines within AD astrocytes. These data suggest that, basally, AD astrocytes present a pro-inflammatory profile.

### Stimulated hiPSC-derived AD astrocytes present an exacerbated pro-inflammatory cytokine response

In response to a CNS insult, microglia and astrocyte communicate to exert a coordinated response to restore brain homeostasis. In an inflammatory environment, microglial activation is essential to unleash astrocytic reactivity [43, 44]. In the context of AD, microglia-astrocyte interactions orchestrate the inflammatory response to pathology, which seems to be both beneficial and detrimental depending on disease status [45]. To mimic astrocytic reactiveness to an inflammatory event and the interaction with microglia, we incubated generated astrocytes with IL-1a, TNF-a and C1q molecules (TIC) for 24 h and assessed their reactive production of pro- and anti-inflammatory cytokines, chemokines and growth factors. As expected and can be seen in Fig. 6C, CTRL astrocytes expressed higher levels of most of the pro-inflammatory cytokines when exposed to TIC conditions. Noteworthy, the response obtained in AD astrocytes was in the same direction but much more increased, showing increased expression in hundreds and even thousands of times in comparison with unstimulated conditions (IL1b: 1151-fold increase, *p* < 0.05; IL-6: 616-fold increase, *p* = 0.06; IL-8: 805-fold increase, *p* < 0.05; IL-23 A: 28-fold increase, *p* < 0.05; CCL-2: 8.7-fold increase, *p* < 0.05; CCL5: 3337-fold increase, *p* < 0.05; TNF-a: 180-fold increase, *p* < 0.05), showing notable and significant higher levels of pro-inflammatory cytokines after TIC incubation in comparison with CTRL astrocytes. Overall, these results suggest that, although presenting moderately higher levels of pro-inflammatory cytokines in unstimulated conditions, in an inflammatory environment, AD astrocytes may trigger an exacerbated response to different stimuli which may compromise their functionality.

### Senescent astrocytes accumulate in AD brains

**Fig. 7:**
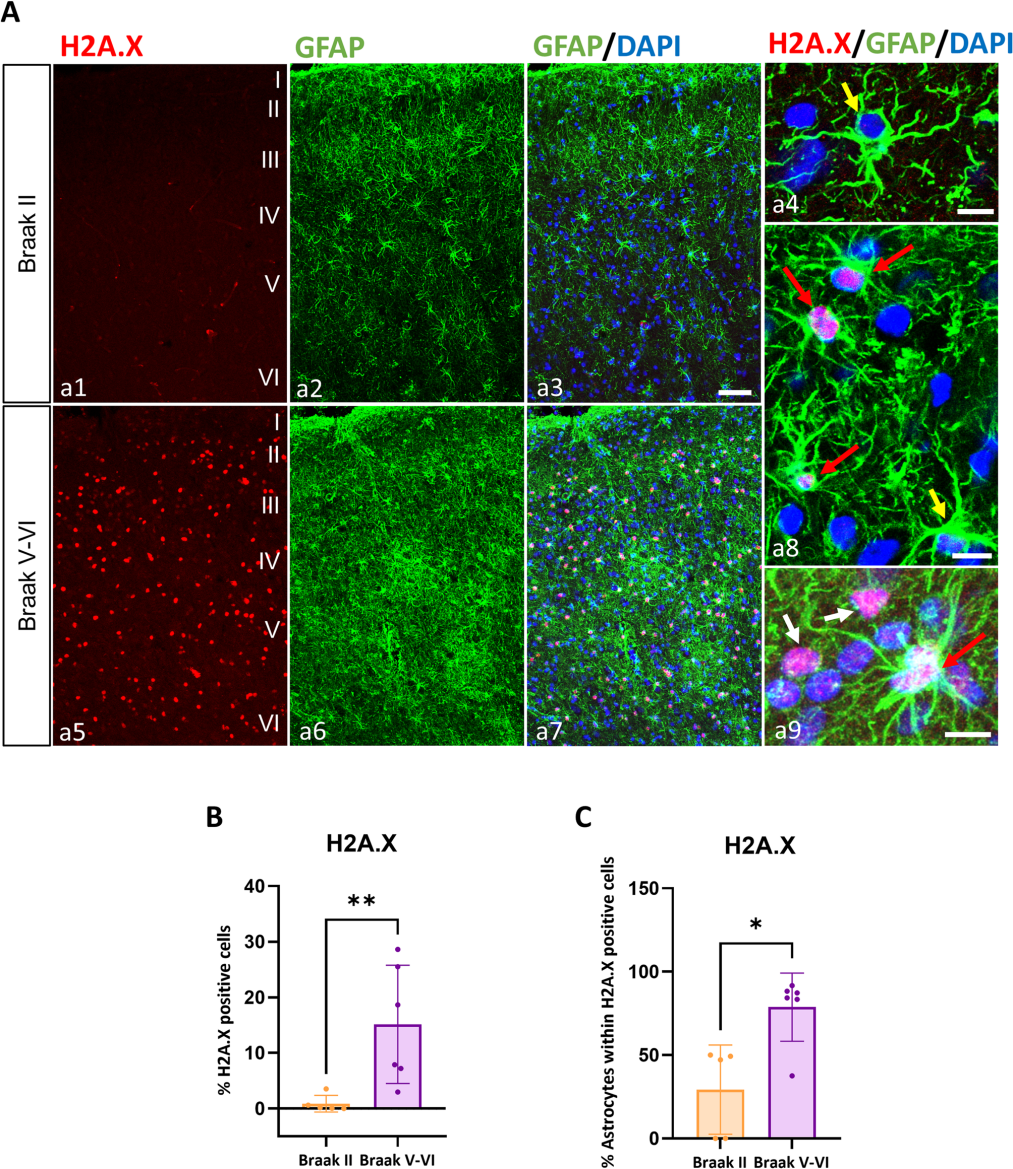
Astroglial senescence in human AD brain. (**A**) Representative immunofluorescence images obtained by confocal microscopy of the senescence marker H2A.X and the astrocytes marker GFAP in frontal cortex samples of Braak II (a1-a4) and Braak V-VI AD (a5-a9) individuals. Details of GFAP-expressing cells not positive for H2A.X (non-senescent astrocytes, yellow arrows) (a4, a8), GFAP-expressing cells positive for H2A.X (senescent astrocytes, red arrows) (a8, a9), and H2A.X-positive cells not expressing GFAP (non-astroglial senescent cells, white arrows) (a9) are shown. Nuclei stained blue with DAPI. Scale bars: a1-a3, a5-a7, 50 μm; a4, a8, a9, 10 μm. a1-a3, a5-a7 and a9 represent the maximum intensity, a4 and a8 represent optical sections. I-VI: cortical layers. (**B**) Immunofluorescence quantification of the percentage of positives cells expressing H2A.X and (**C**) the percentage of astrocytes within these H2A.X positives cells in Braak II (*n* = 5) and Braak V-VI (*n* = 6) subjects. The values of each individual are represented, together with the mean and standard deviation. *p < 0.05, **p < 0.01

Since our results indicated that hiPSC-derived astrocytes from AD patients show a senescent state, we wanted to test whether this phenomenon was also occurring in the brain of AD patients. Therefore, we evaluated (Fig. 7 A a1-a9) the presence of the senescence-associated DNA damage marker H2A.X together with the astrocytic marker GFAP in postmortem human brain samples (frontal cortex) from AD patients (Braak V-VI) compared to age-matched individuals without neurological deficits (Braak II). We observed a significant increase in the percentage of senescent cells in Braak V-VI AD patients compared to Braak II cases (17.55-fold increase, 15.13 ± 10.64% vs. 0.86 ± 1.5%, *p* < 0.01) (Fig. 7B). Furthermore, among the senescent cells, 78% were astrocytes (GFAP+) in AD patients, while only 29% were astrocytes in Braak II subjects (2.69 fold increase, 78.77 ± 20.40% vs. 29.28 ± 26.75%, *p* < 0.05) (Fig. 7 C). These results were consistent with those obtained in hiPSC-derived astrocytes and indicate a remarkable increase in senescent astrocytes in patients, which may be key in driving the pathological process of AD. Furthermore, they highlight the suitability of the hiPSC-derived model for the study of this neurodegenerative disease.

### hiPSC-derived AD astrocytes provide deficient neuronal support

**Fig. 8:**
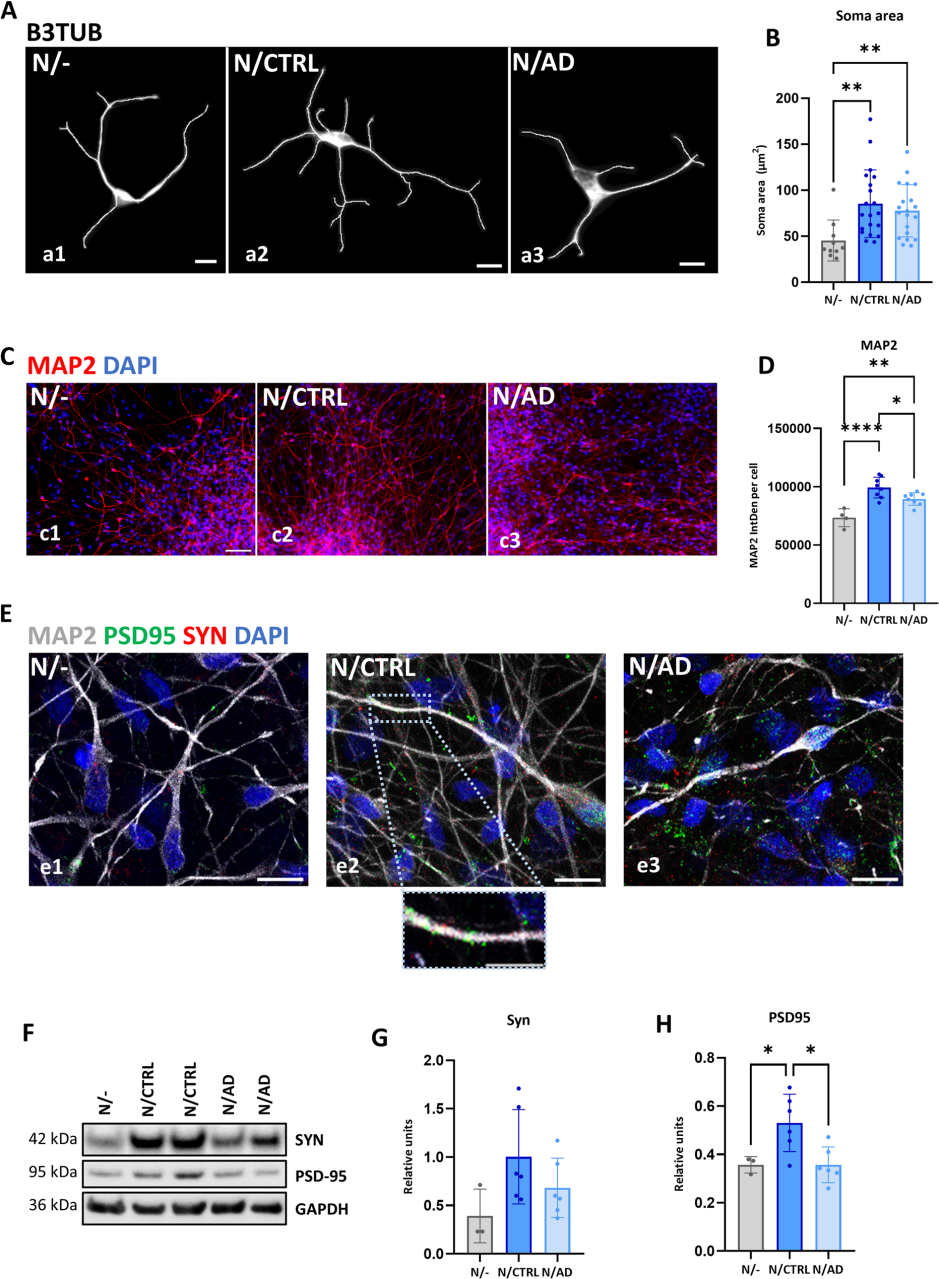
Neuronal support capacity of human iPSC-derived AD astrocytes. (**A**) Representative images of human CTRL iPSC-derived neurons (N) after 3 days in coculture without astrocytes (N/-; a1) and in the presence of iPSC-derived CTRL (N/CTRL; a2) or AD (N/AD; a3) human iPSC-derived astrocytes, obtained by immunofluorescence for β3 tubulin, and subsequently binarized with FIJI. Nuclei stained blue with DAPI. 10–20 individual neurons analyzed per condition. Scale bar: 10 μm. (**B**) Quantification of the area of ​​the soma of neurons in the three culture conditions studied. (**C**) Representative immunofluorescence images for MAP2 (red) under the different coculture conditions: without astrocytes (N/-; c1) and in the presence of CTRL (N/CTRL; c2) or AD (N/AD; c3) human iPSC-derived astrocytes. Nuclei stained blue with DAPI. Scale bar: 50 μm. (**D**) Quantification of MAP2 fluorescence intensity per cell in neuron cultures under the three conditions analyzed. (**E**) Representative confocal microscopy images of the triple immunofluorescence for the dendritic marker MAP2 (white) and the synaptic markers PSD95 (green) and Syn (Synaptophysin) (red) in the different coculture conditions, without astrocytes (N/-; e1) and in the presence of CTRL astrocytes (N/CTRL; e2) or AD (N/AD; e3) human iPSC-derived astrocytes. Nuclei were counterstained in blue with DAPI. Scale bars: 10 μm. Below, higher magnification image of a detail of a neuronal extension immunostained for these synaptic proteins. Scale bar: 5 μm. (**F**) Western blot analysis for the synaptophysin and PSD95 markers in the different coculture conditions. (**G**) Quantification of the expression of Syn and (**H**) PSD95 markers by western blot. Values ​​relative to GAPDH. The individual values ​​of each condition (per replicate) are represented, together with the mean and standard deviation. *p < 0.05, **p < 0.01, ***p < 0.001, ****p < 0.0001

As the main function of astrocytes is to participate in neuronal circuits development and to provide support to neurons to be able to maintain proper functioning [46], we evaluated whether the acquisition of this senescent phenotype within AD astrocytes may affect their capacity of providing neuronal support. To assess that, we co-cultured generated astrocytes with hiPSC-derived early neurons for up to 4 weeks and performed several determinations at different time points. Just after 3 days of co-culture, we assessed the morphology of individual neurons (immunolabeled for beta-3 tubulin) cultured alone, co-cultured with CTRL astrocytes or with AD astrocytes (Fig. 8A a1-3). Although we did not measure significant differences related to neuronal complexity (not shown), we found that those neurons co-cultured with astrocytes presented a higher neuronal soma area, especially those neurons co-cultured with CTRL astrocytes (Fig. 8B). As the neuronal soma is the cell compartment where most organelles concentrate and most of the protein translation takes place, a higher cell soma just after 3 days of co-culture may be indicative of neurons with higher capacity of further maturation.

In fact, when we assessed MAP2 (microtubule-associated protein 2) expression (a mature neuronal marker, as a proxy to neuronal maturation) after 4 weeks of co-culturing the cells (Fig. 8C c1-3) alone, with CTRL astrocytes or with AD astrocytes, we observed a significant increase (35%) in the expression of this marker when the neurons were cultured together with astrocytes, but with significant differences depending on the source of astrocytes, as AD astrocytes produced lower increase in the expression of this marker (12% reduction, *p* < 0.05, Fig. 8D) in comparison with control astrocytes.

Finally, to evaluate whether astrocytes influenced synapse formation, we assessed by triple immunofluorescence and WB in these cultures the presence of presynaptic (synaptophysin) and post-synaptic (PSD95, postsynaptic density protein 95) markers in MAP2-positive dendrites (Fig. 8E e1-3). As can be seen in Fig. 8F-H, a higher expression of both markers, especially of PSD95, was found when neurons were cultured together with control astrocytes, but not when they were cultured alone or in the presence of AD astrocytes. Together, these data suggest that senescent AD astrocytes provide a significant deficient neuronal support in comparison with the astrocytes derived from cognitively healthy subjects.

## Discussion

In AD, reactive astrocytes are present from early disease stages [14, 47] and are associated with the pathology and progression of the disease [48]. In fact, they are closely located to Ab plaques, attempting to eliminate presynaptic dystrophies and preserve proper brain functioning [49]. In addition to be affected by the growing AD pathology, astrocytes in AD present a reactive profile losing the expression of supporting genes [13, 50]. Recent evidences support the idea that different reactive astroglial states coexist in the AD brain [15] and their signatures changes along AD continuum and brain regions [51].

In our study, we aimed to address whether metabolism is intrinsically affected in AD astrocytes. For that, we generated hiPSC lines from AD patients and age-matched individuals with unaffected cognition. To assure the presence of a robust AD phenotype, we selected AD patients carrying in homozygosis the *APOE4* variant, the strongest genetic risk factor contributing to the development of late-onset AD [52] and recently proposed as a familial form of the disease [19]. Not previously described in human models, employing a serum-free protocol (which maintains the cells in a steady state) we observed that AD *APOE4/4* astrocytes have an increased mitochondrial fusion, which co-localize with phagosomes around the perinuclear area. As a general mechanism, damaged mitochondria (characterized by having lower membrane potentials and higher accumulation of ROS) are excised by mitochondrial fission and degraded by mitophagy in the perinuclear area to maintain mitochondrial homeostasis [53]. The accumulation of mitochondria in the perinuclear area coupled with phagosomes we observed in disease astrocytes (and also recently described for sAD astrocytes [54]), might fit with this assumption, although we did not observe an increased expression of proteins involved in mitophagy. On the other hand, despite presenting much higher levels of mitochondrial ROS, we observed that in AD astrocytes mitochondrial fission was not enhanced but inhibited, in favor of mitochondrial fusion, evidenced by the presence of particularly elongated mitochondria in AD astrocytes. In this sense, mitochondrial fusion is promoted when metabolic requirements are high, to sustain ATP biosynthesis in the early steps of several energy-demanding processes [55].

In aging and neurodegenerative conditions, the brain acquires a hypometabolic state which it is thought to be an important contributor to neurodegeneration [56, 57]. In AD, astrocytes present a hypometabolic profile which compromise their functioning [54, 58]. In fact, a reduction in the oxidative phosphorylation capacities have been observed for hiPSC-derived astrocytes from sAD patients and also harboring *APOE4* [59, 60]. Regarding the metabolic parameters of our AD astrocytes, we did not observe the expected reduction in the basal metabolism in these cells but a significant increase in all the parameters associated to mitochondrial respiration and glycolysis, accompanied by significantly higher ROS levels. One possible explanation for our observations is that these disease-specific astrocytes, even when cultured in basal conditions, develop a high energy-demanding phenotype, as a consequence of acquiring a cellular senescence state [55], which require a high metabolic activation to sustain it. This might explain why AD astrocytes, despite producing higher mitochondrial ROS and displaying abnormal mitochondrial spatial distribution, exhibited high metabolic rates suggestive of accelerated mitochondrial oxidative phosphorylation and glycolysis. The much higher consumption of glutamate we observed within AD astrocytes (Supp. Fig. 6) may well be related to the exacerbated metabolism displayed by these cells, as glutamate can be used as a source of energy by astrocytes [61], which may interfere with their capacity of recycling this neurotransmitter.

Cellular senescence, originally defined as loss of replicative potential, leads to notable cellular alterations which compromise their functionality [39]. Senescent cells accumulate with aging, and this accumulation is a physiologically important driver of age-associated functional decline [62]. In fact, cellular senescence has been proposed as an important contributor to the progression of AD [63, 64], and glial senescence seems to be an essential driver [65]. Among the different cellular processes involved, mitochondrial dysfunction has been proposed as a main driver of cellular senescence [38]. Our results can be explained by the fact that mitochondrial fusion increases, the mitophagy process decreases, and energetic metabolism increases during cellular senescence [38, 39, 41]. Although in our AD astrocytes the mitophagic pathway was not reduced compared to the control group but equalized in expression, considering the higher mitochondrial content in AD astrocytes, a lower activity of this mitochondrial degradation mechanism can actually be suggested. Together, these phenomena may drive the acquisition of a senescent phenotype, which was finally confirmed since these cells accumulated lipid droplets, had a lower proliferative capacity, display cellular hypertrophy, significantly upregulated senescent markers and developed a SASP secretory phenotype (characterized by the expression of proinflammatory cytokines) in basal conditions.

Sustained neuroinflammation is thought to be one of the mechanisms driving AD pathology, which may be influenced by the accumulation of glial senescent cells [38, 65]. A recent study in AD brains using single nuclear transcriptomics identified a cumulative presence of senescent glial cells with disease progression [66]. Hu and Yanling pointed out that the emergence of senescent microglia in AD was due to sustained proliferation in response to AD pathology [67]. Also, the presence of senescent oligodendrocyte precursor cells have been reported in AD models and patient brains [68]. The presence of senescent astrocytes in AD have been poorly characterized and associated mostly to tau pathology [69, 70]. To validate our findings in hiPSC-derived astrocytes, we evaluated the presence of senescent astrocytes in AD brains, finding a notable presence of phosphorylated H2A.X^+^ cells in the frontal cortex of Braak V-VI AD patients, which represented the 15% of all cells in this brain area, with most of them (78%) being GFAP^+^ astrocytes. As the presence of phosphorylated H2A.X is indicative of DNA breaks and a proxy to cell senescence, the notable presence of senescent astrocytes in AD brain cortex (and almost absent in age-paired controls), may suggest that astrocytic senescence may be key in AD pathology.

Employing our hiPSC-derived model, we evaluated the functional consequences of this phenomenon. Probably, the main functionality altered by cellular senescence is the acquisition of a SASP, which alters the communication of cells with their environment [42]. This is especially important for astrocytes as, together and in communication with microglia, regulate brain homeostasis [5]. To model the crosstalk that astrocytes receive from microglia under pro-inflammatory conditions, we incubated them with IL-1α, TNFα and C1q [43], observing an exacerbated and pro-inflammatory response by those astrocytes derived from AD patients. Therefore, the senescent phenotype of AD astrocytes may be related to a greater proinflammatory response of these cells. Since a lower neuronal support capacity of these astrocytes was evidenced, this study confirms that senescent and pro-inflammatory AD astrocytes may contribute to neurodegeneration. In fact, senescent astrocytes induce neuronal excitotoxicity [71]. Although further analysis in this regard would be necessary to confirm this, these results reinforce the direct relationship between astroglial pathology and neurodegeneration in AD.

We observed notable mitochondrial alterations present in AD astrocytes. The lack of mitochondrial homeostasis may lead to the disruption of mitochondrial integrity and unleashing of mitochondrial DNA into the cytoplasm, activating DNA sensing mechanisms as the cGAS-STING signaling, strongly associated to aging [72, 73] and neurodegenerative conditions [74–77]. This pathway, preferentially described in microglia, it is also expressed in astrocytes [78–80]. The activation of this pathway leads to the acquisition of a pro-inflammatory profile and the production of SASP. As a possible explanatory mechanism, we speculate that in AD *APOE4/4* astrocytes, mitochondrial oxidative stress could be activating this pathway and thus inducing a senescent and proinflammatory phenotype in AD astrocytes.

As previously outlined, different reactive astrocytic phenotypes are present in AD brains [15]. Meanwhile most of studies have classified these altered phenotypes as reactive phenotypes [14], it is plausible that some of these studies have actually described astrocytic senescence and the accompanying phenotype. Proper definition of this astrocytic senescent phenotype and its functional consequence is necessary to fully address the role that this phenomenon has in the disease. In this sense, senolytic drugs (efficacious in preclinical studies and currently in clinical trials for AD) have the potential to modify the balance of senescent cells in the AD brain [81].

## Conclusions

In conclusion, astrocytes generated from hiPSCs of AD patients with *APOE4/4* genotype present dysfunctional characteristics associated with human pathology at the mitochondrial, metabolic and pro-inflammatory reactive levels, which are linked with the acquisition of a senescent phenotype, a phenomenon also occurring in AD brains. Furthermore, this senescent and pro-inflammatory state compromises astrocytic capacity of neuronal support, which may be directly related to neurodegeneration. Future efforts aiming to clarify the degree and contribution of senescent astrocytes to AD pathology are mandatory to develop effective drugs able to counteract the course of this complex disease.

## Study limitations

One of the main limitations of the present study is the inability to address whether alterations found in disease astrocytes are due to intrinsic AD alterations or the consequence of bearing the *APOE4/4* genotype due to the lack of isogenic lines. Studies performed in gene-edited PSC lines and targeted-replacement mice have reported intrinsic features in *APOE4* astrocytes, especially lipid dysregulation [82–84], changes in the inflammatory profile [85] and impaired contribution to the blood brain barrier [86]. The fact this is the first study with hiPSC-derived astrocytes reporting astrocytic senescence may indicate that both factors (harboring the *APOE4/4* genotype plus developing the disease) may contribute to the acquisition of this cell state. In any case, further studies employing isogenic controls from both control and AD hiPSC lines would be needed to fully understand the causes and consequences of the predisposition of AD astrocytes to adopt a senescent phenotype to evaluate a treatment strategy able to counteract this phenomenon.

Our study reveals that under basal conditions, astrocytes derived from *APOE4/4* AD patients present a senescent phenotype which compromises their functionality and this is also present in the AD brain. How specifically this affects astrocytic capacity of preserving brain homeostasis and its contribution to AD progression and neurodegeneration should be the target of further research.

## Supplementary Information

Supplementary Material 1.

Supplementary Material 2.

## Data Availability

All data generated or analyzed during this study are included in this published article and its supplementary information files. Data and resources generated throughout the present work are available to other investigators under reasonable requests.

## Abbreviations

Ab: amyloid-beta
AD: Alzheimer´s disease
APOE: apolipoprotein E
AQP4: aquaporin 4
ATP: adenosine triphosphate
CTRL: control
ECAR: extracellular acidification rate
FACS: flow-activated cell sorting
GFAP: glial fibrillary acidic protein
H2A.X: H2A histone family member X
hiPSCs: human induced pluripotent stem cells
IFN-g: interferon gamma
IL: interleukin
LAMP1: lysosomal associated membrane protein 1
LAMP2: lysosomal associated membrane protein 2
LC3B: autophagy marker light chain 3B
MAP2: microtubule-associated protein 2
OCR: oxygen consumption rate
PSD95: postsynaptic density protein 95
ROS: reactive oxygen species
SASP: senescence-associated secretory phenotype
TNFa: tumor necrosis factor alpha
TOMM20: translocase of outer mitochondrial membrane 20
