# Supplementary material for: Human iPSC-derived APOE4/4 Alzheimer´s disease astrocytes exhibit a senescent and pro-inflammatory state that compromises neuronal support
Source: J Neuroinflammation. 2025 Dec 12;23:9. doi: 10.1186/s12974-025-03607-z (PMC12781401; doi:10.1186/s12974-025-03607-z)
Supplement: Supplementary file 1 — Supplementary Material 1. [file 12974_2025_3607_MOESM1_ESM.pdf]

Supplementary Figures

HUMAN iPSC-DERIVED APOE4/4 ALZHEIMER´S DISEASE ASTROCYTES EXHIBIT A SENESCENT AND PRO-INFLAMMATORY STATE THAT COMPROMISES NEURONAL SUPPORT

Laura Caceres-Palomo, Elisabeth Sanchez-Mejias, Laura Trujillo-Estrada, Juan José Pérez-Moreno, Elba Lopez-Oliva, Tau En Lim, Leah DeFlitch, Serena H. Chang, Lucas Kampman, M. Ryan Corces, Mathew Blurton-Jones, Ines Moreno-Gonzalez, Alberto Pascual, Javier Vitorica, Juan Antonio Garcia-Leon[‡] and Antonia Gutierrez[‡]

APOE

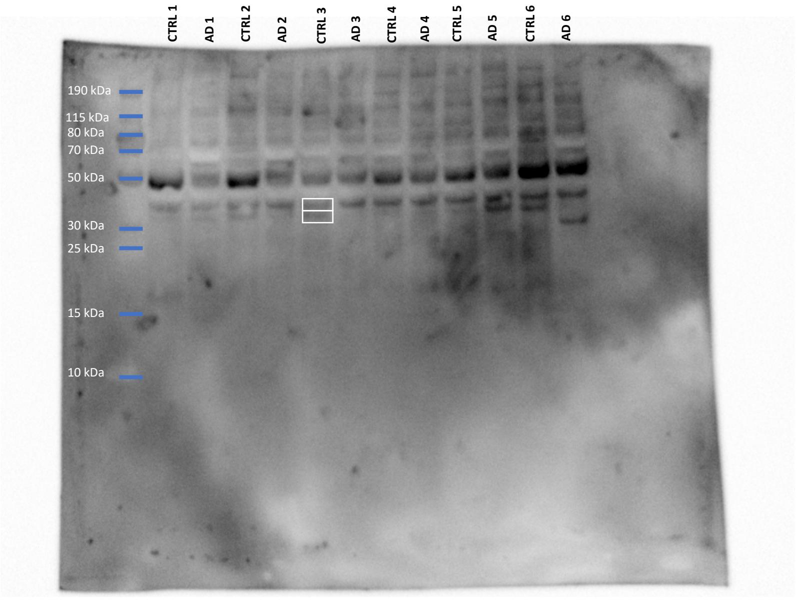

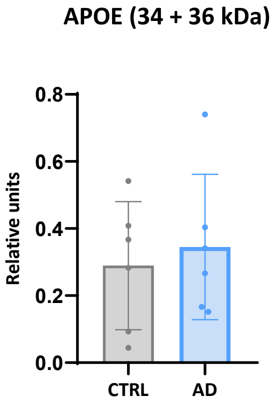

GAPDH

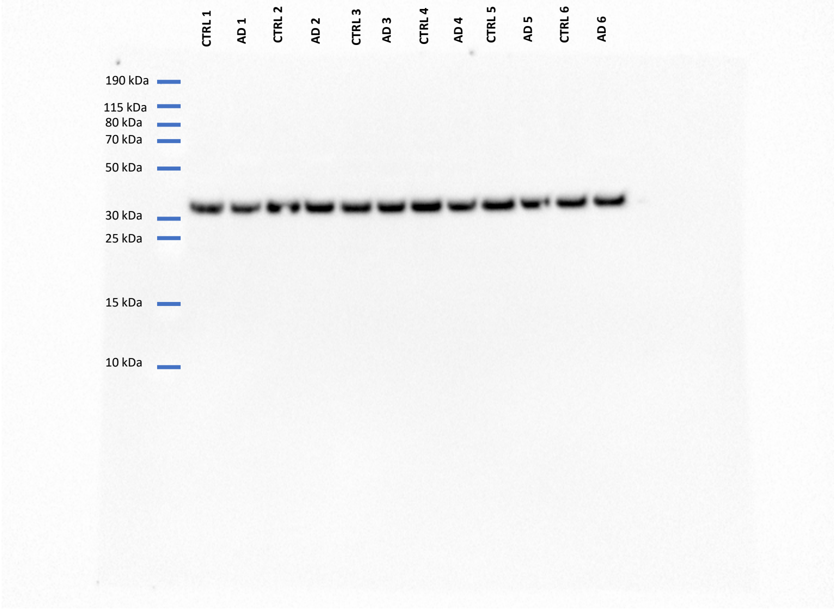

Supplementary Figure 1. APOE quantification in cell extracts by wester blot (WB).

A

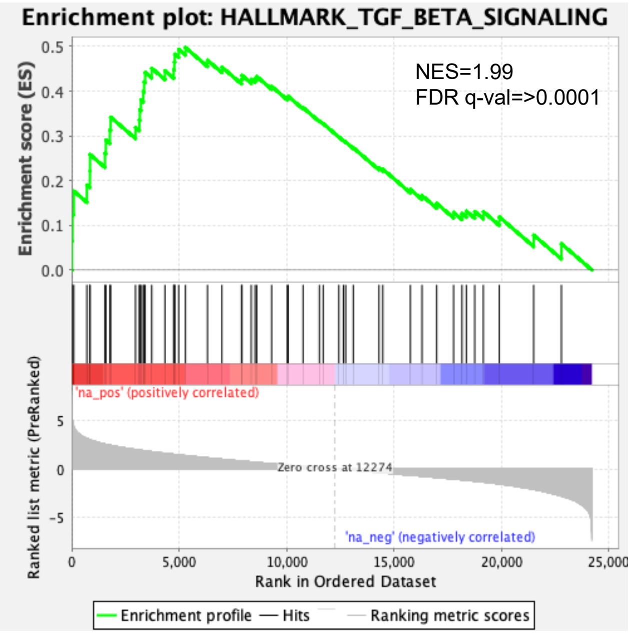

B

Table: GSEA Results Summary

Dataset	AD_vs_CTRL_for_GSEA
Phenotype	NoPhenotypeAvailable
Upregulated in class	na_pos
GeneSet	HALLMARK_TGF_BETA_SIGNALING
Enrichment Score (ES)	0.49750215
Normalized Enrichment Score (NES)	1.9937031
Nominal p-value	0.0
FDR q-value	0.0
FWER p-Value	0.0

C

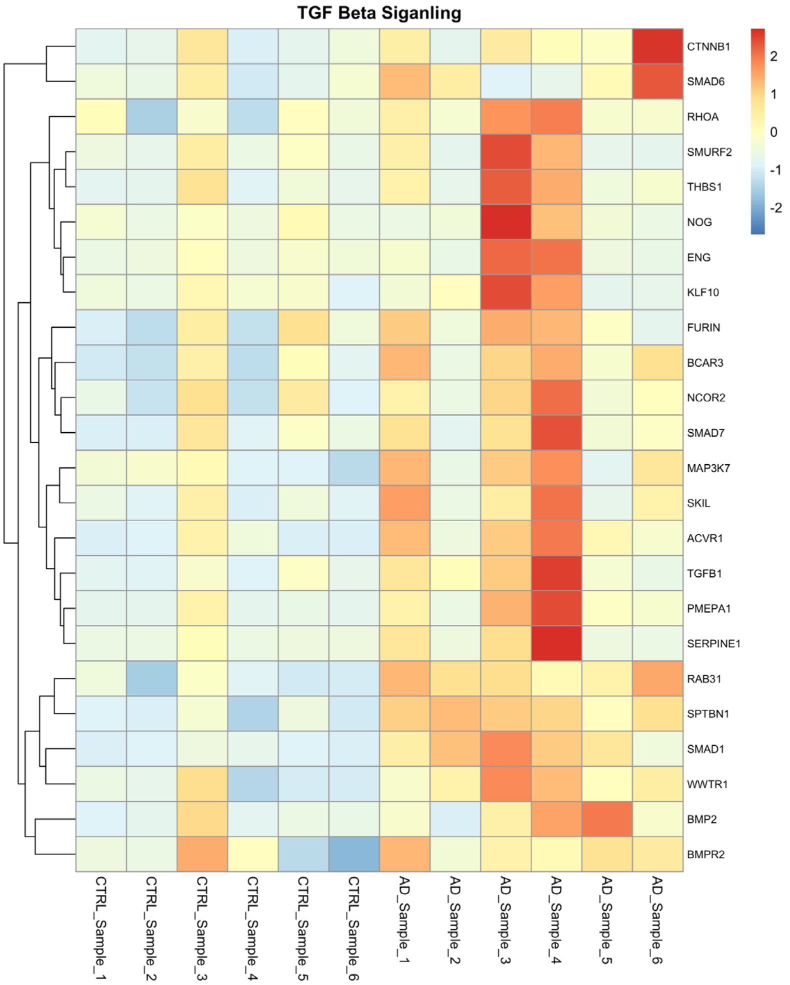

Supplementary Figure 2. Gene set enrichment analysis (GSEA) of the transforming growth factor beta (TGFb) pathway. A) Enrichment plot, B) GSEA results summary and C) heatmaps of the top 24 ranking leading edge genes.

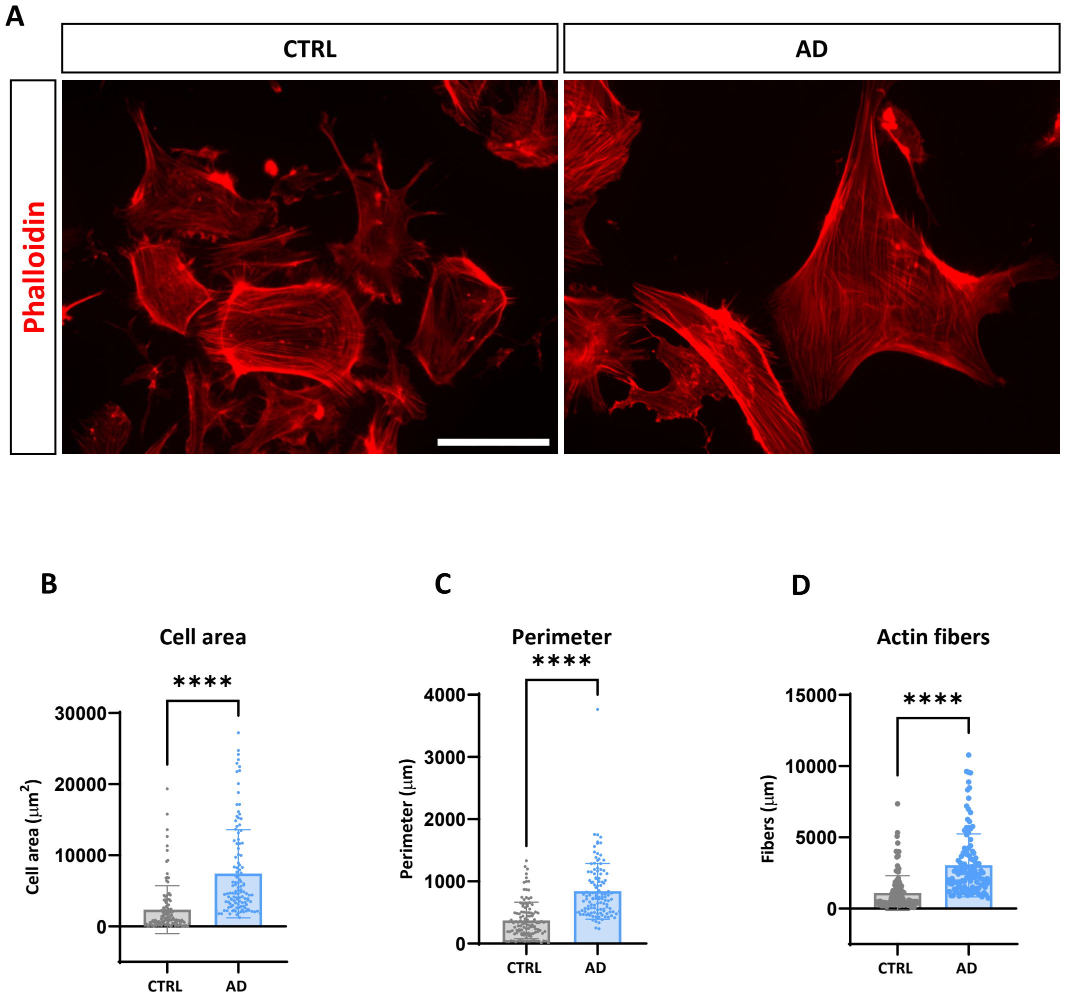

Supplementary Figure 3. Morphological characterization by Phalloidin staining of astrocytes. A) Representative fluorescence images of Phalloidin staining of cytoskeletal actin filaments in CTRL and AD astrocytes. Scale bar: 100 µm. Quantification of astroglial cell area (B), perimeter (C), and total actin fiber length (D) of astrocytes. Individual cell values are represented, along with the mean and standard deviation.

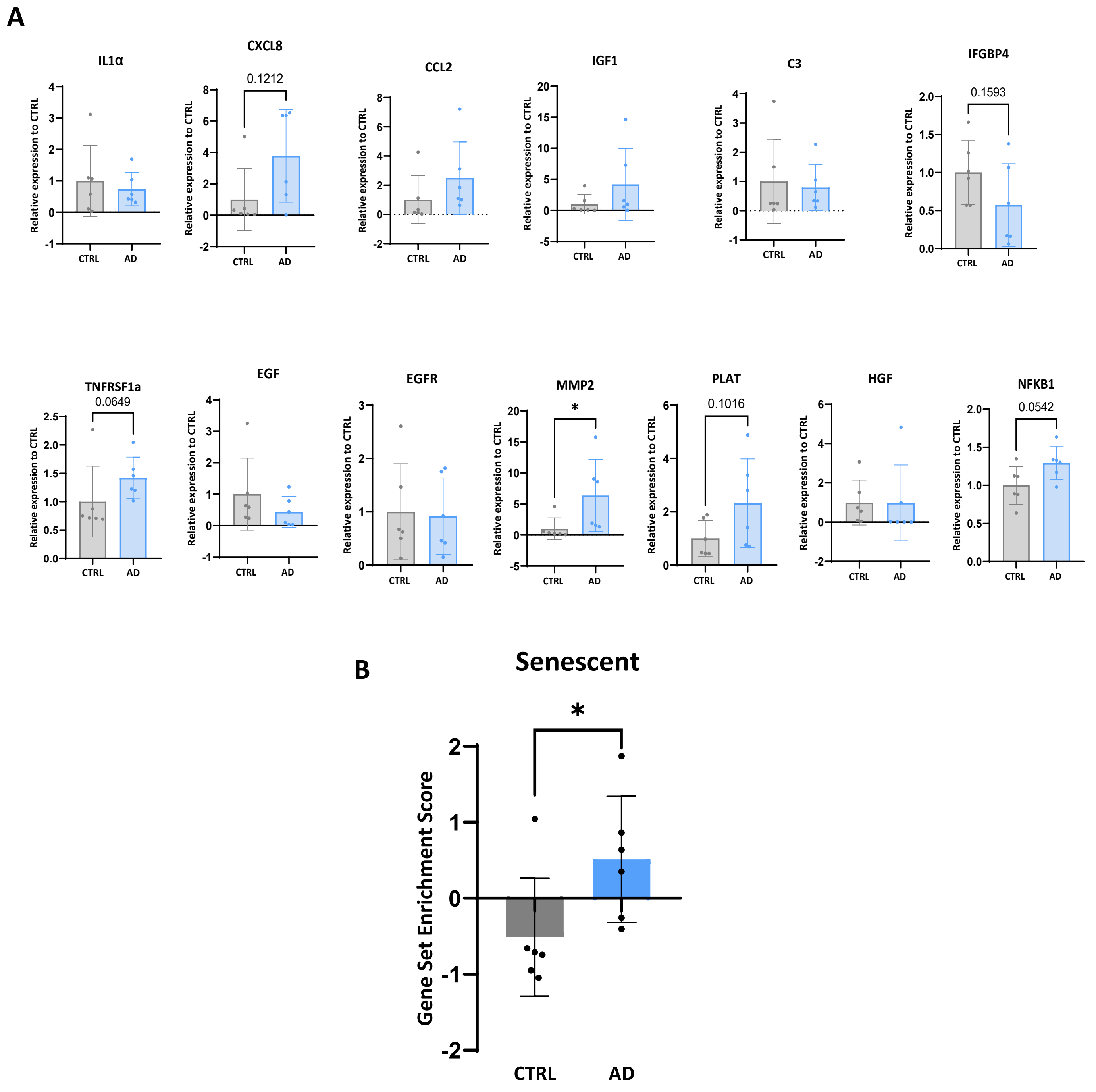

Supplementary Figure 4. Expression of senescent markers by human iPSC-derived AD APOE4/4 astrocytes. A) Analysis of the expression by qPCR of the set of genes most influencing the acquisition of a senescent phenotype (Saul D. et al. 2022). B) Gene set score analysis of the above senescence genes. Individual values for each cell line are represented, along with the mean and standard deviation. *p<0.05.

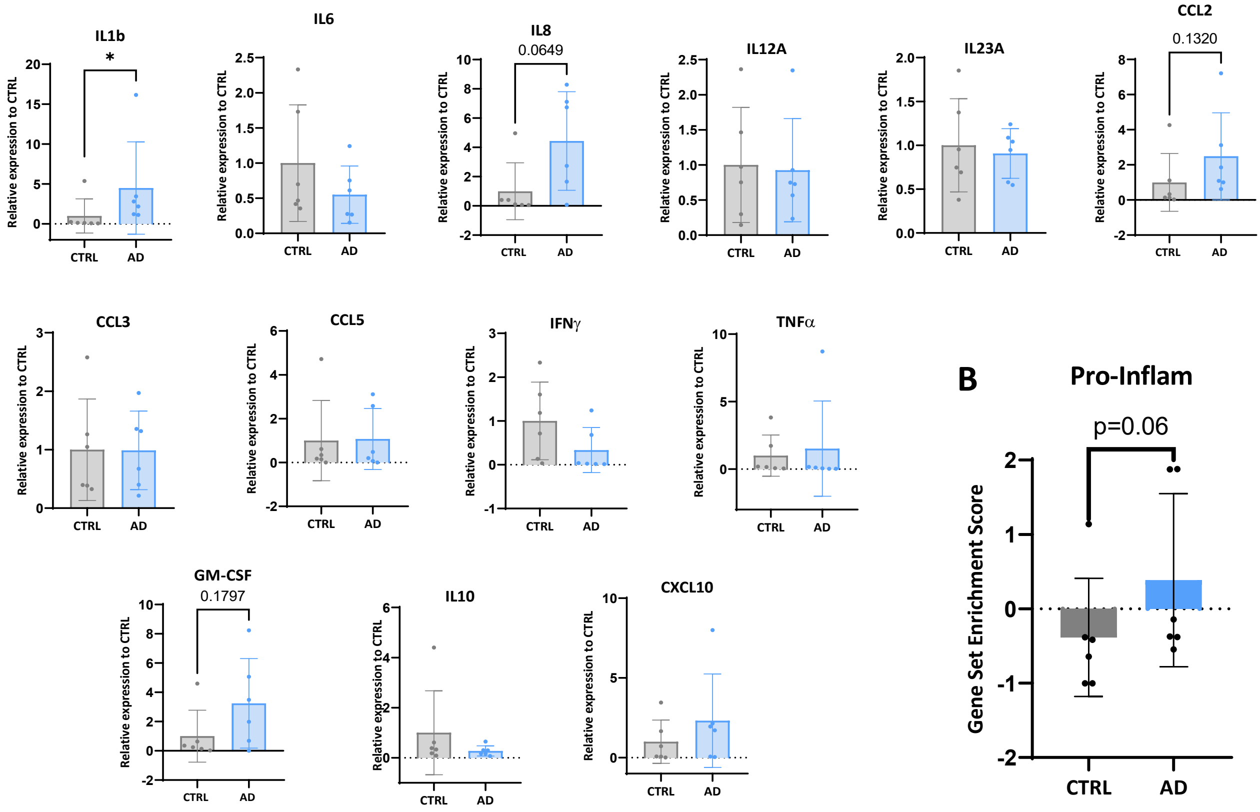

Supplementary Figure 5. Expression of inflammatory cytokines and chemokines by human iPSC-derived AD APOE4/4 astrocytes. A) Evaluation by qPCR of the gene expression of several pro- and anti-inflammatory cytokines and chemokines under basal conditions in CTRL and AD astrocytes (IL1β, IL6, IL8, IL12A, IL23A, CCL2, CCL3, CCL5, IFNγ, TNFα, GM-CSF, IL10 and CXCL10). B) Gene score analysis of the main proinflammatory genes (IL1β, IL6, IL8, IL12A, IL23A, CCL2, CCL3 CCL5, IFNγ, TNFα). B) Gene set score analysis of the above senescence genes. Individual values for each cell line are represented, together with the mean and standard deviation.

A

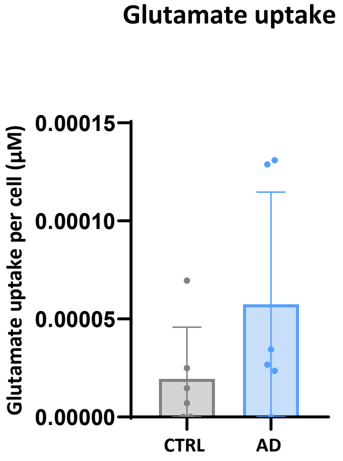

B

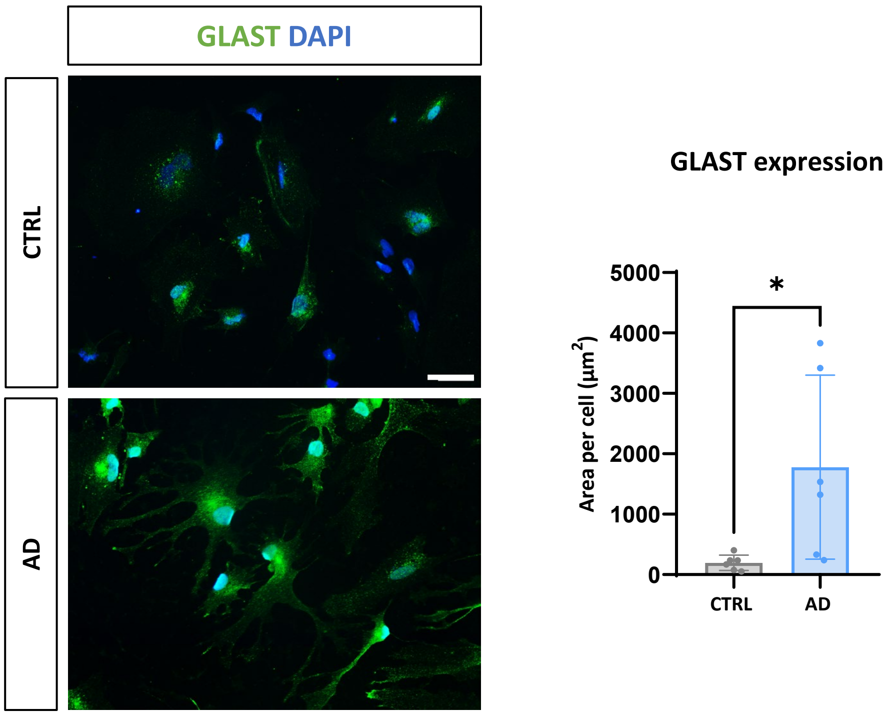

Supplementary Figure 6. Glutamate uptake by astrocytes. A) Quantification of glutamate consumption per cell by astrocytes from the two experimental groups. B) Images and quantification of the expression of the glutamate transporter GLAST in the two study groups. Nuclei were stained blue with DAPI. Scale bar: 50 µm. Individual values for each cell line are represented, along with the mean and standard deviation.

WESTERN BLOTS

WB1

LAMP1

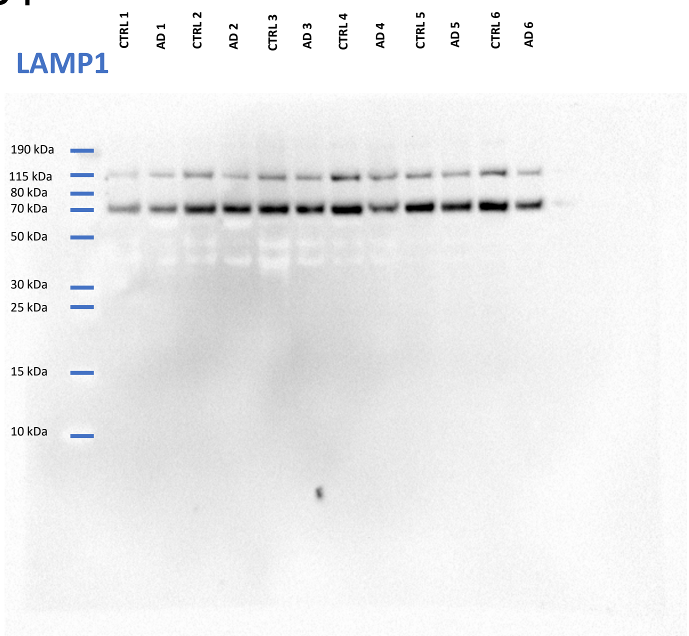

GAPDH

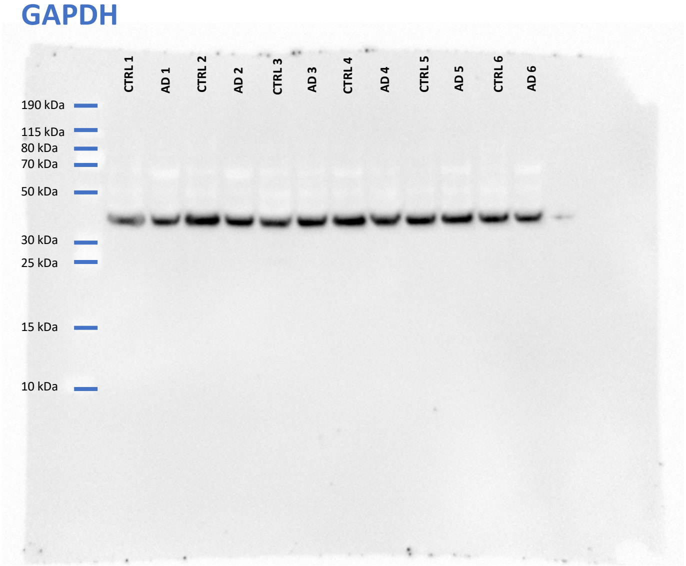

LC3B

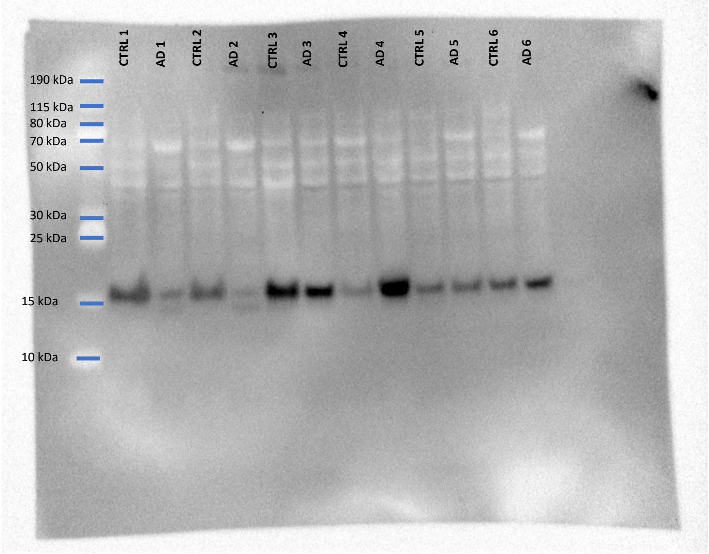

WB2

PINK1

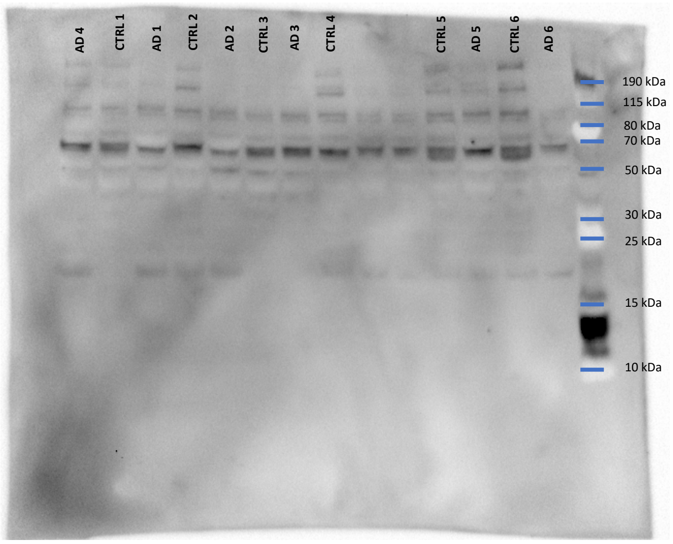

OPA1

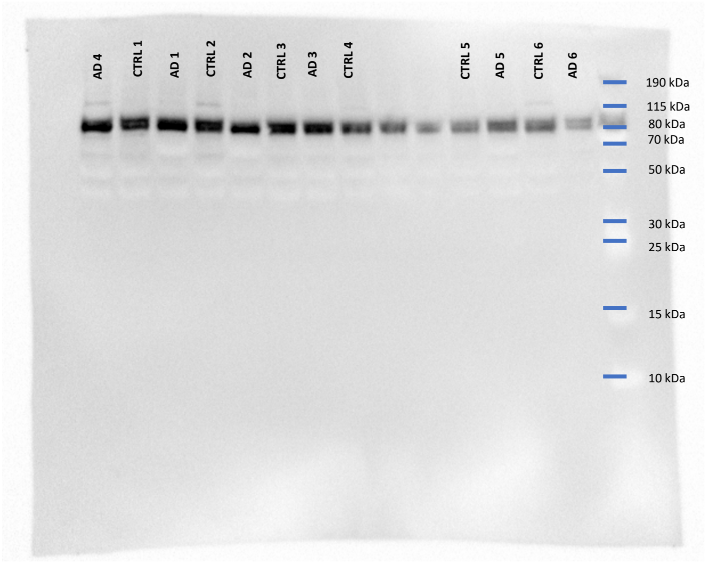

Mfn2

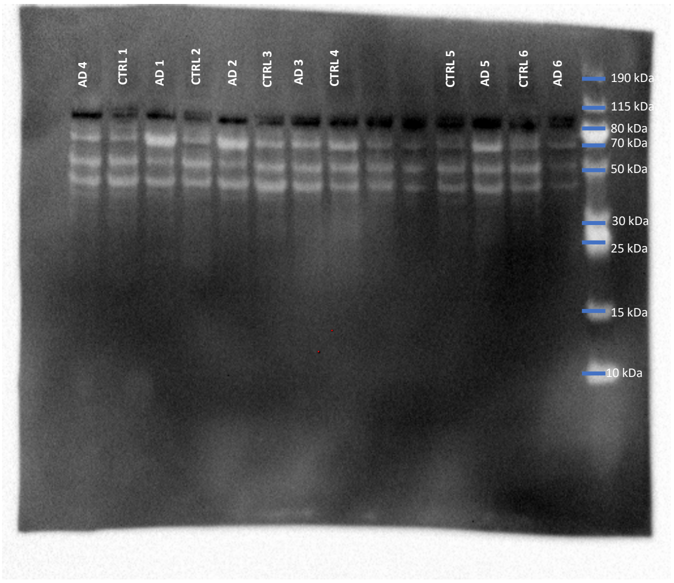

GAPDH

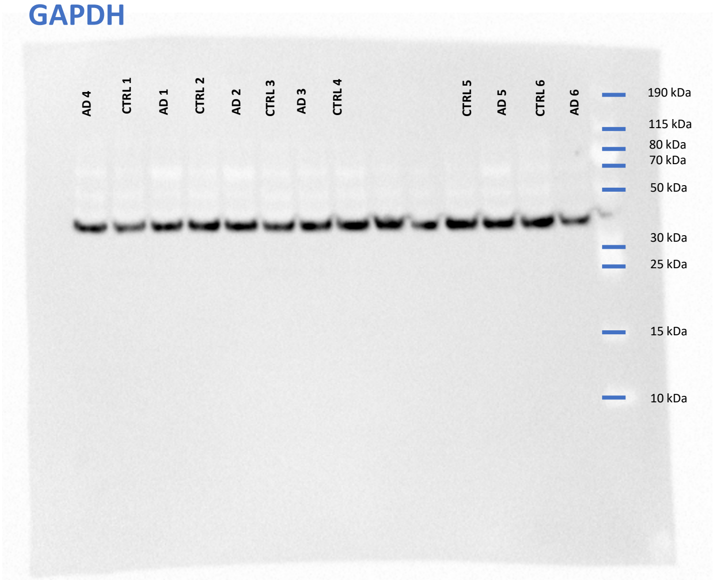
AD 4
CTRL 1
AD 1
CTRL 2
AD 2
CTRL 3
AD 3
CTRL 4
CTRL 5
AD 5
CTRL 6
AD 6
190 kDa
115 kDa
80 kDa
70 kDa
50 kDa
30 kDa
25 kDa
15 kDa
10 kDa

WB3

DRP1

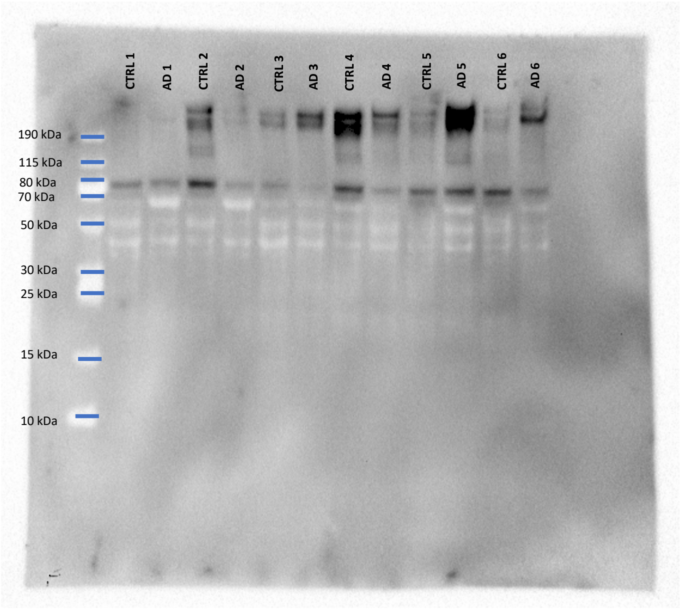

PARK2

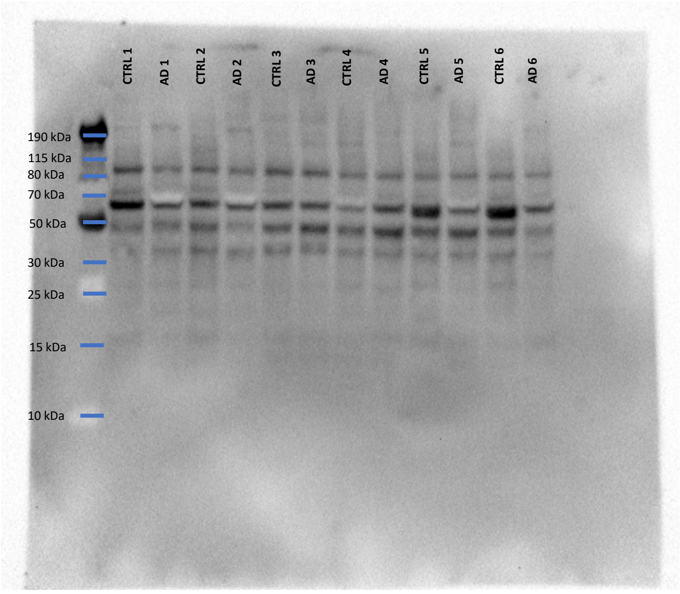

GAPDH

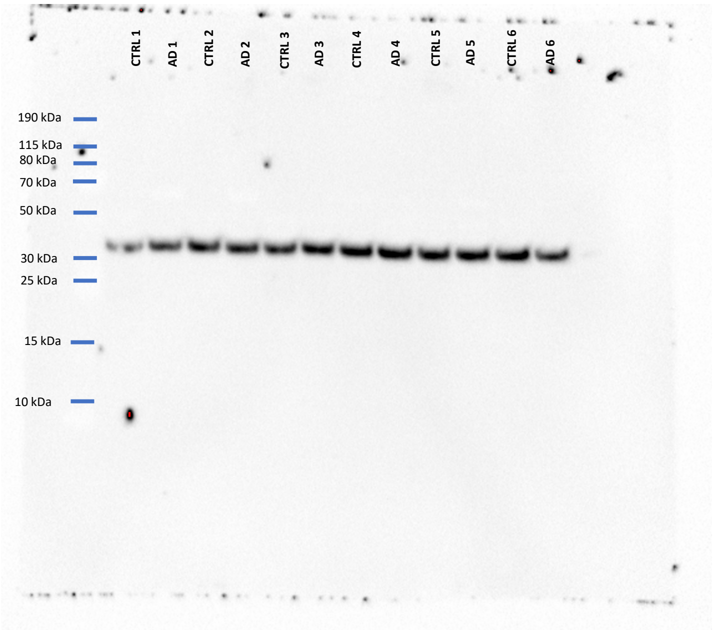

WB4

Lamp2

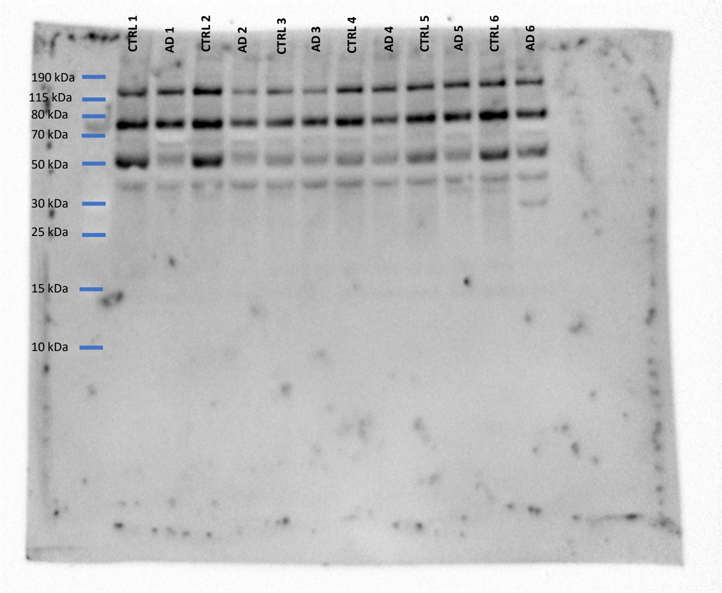

GAPDH

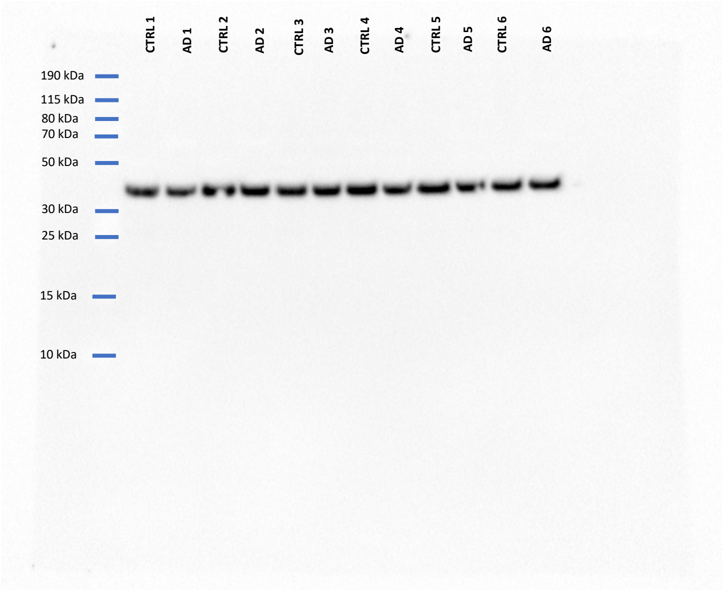

WB5

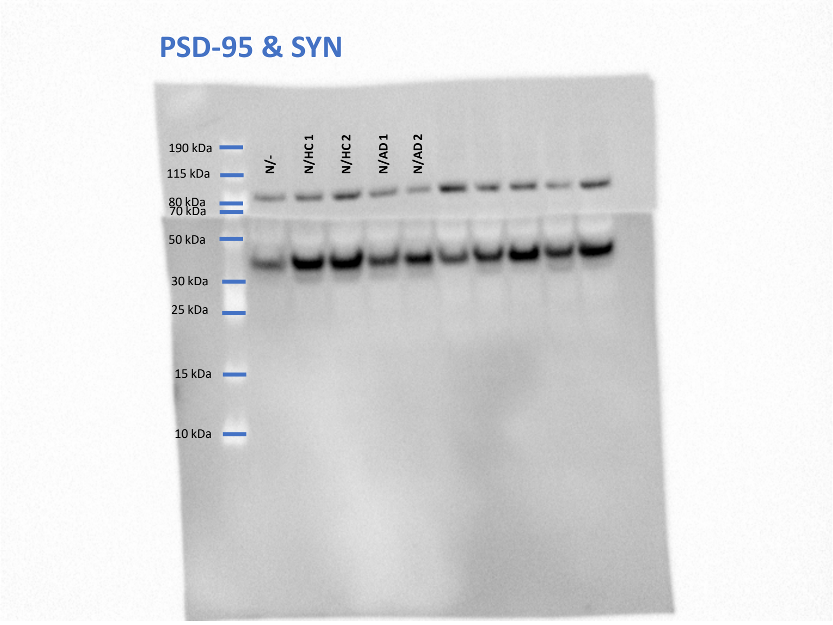
PSD-95 & SYN
190 kDa
115 kDa
80 kDa
70 kDa
50 kDa
30 kDa
25 kDa
15 kDa
10 kDa
N/-
N/HC1
N/HC2
N/AD1
N/AD2

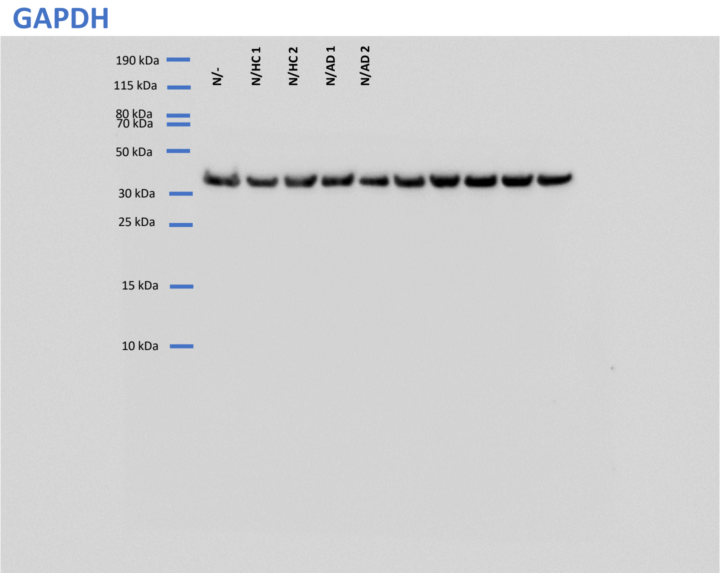
GAPDH
190 kDa
115 kDa
80 kDa
70 kDa
50 kDa
30 kDa
25 kDa
15 kDa
10 kDa
N/-
N/HC1
N/HC2
N/AD1
N/AD2
