# Supplementary material for: Human iPSC-derived APOE4/4 Alzheimer´s disease astrocytes exhibit a senescent and pro-inflammatory state that compromises neuronal support
Source: J Neuroinflammation. 2025 Dec 12;23:9. doi: 10.1186/s12974-025-03607-z (PMC12781401; doi:10.1186/s12974-025-03607-z)
Supplement: Supplementary file 2 — Supplementary Material 2. [file 12974_2025_3607_MOESM2_ESM.pdf]

Supplementary Information

HUMAN iPSC-DERIVED APOE4/4 ALZHEIMER´S DISEASE ASTROCYTES EXHIBIT A SENESCENT AND PRO-INFLAMMATORY STATE THAT COMPROMISES NEURONAL SUPPORT

Laura Caceres-Palomo, Elisabeth Sanchez-Mejias, Laura Trujillo-Estrada, Juan José Pérez-Moreno, Elba Lopez-Oliva, Tau En Lim, Leah DeFlitch, Serena H. Chang, Lucas Kampman, M. Ryan Corces, Mathew Blurton-Jones, Ines Moreno-Gonzalez, Alberto Pascual, Javier Vitorica, Juan Antonio Garcia-Leon‡ and Antonia Gutierrez‡

Media related to experimental procedures

Table 1. Neural induction medium.

Reagent	Final concentration	Supplier and reference
**DMEM/F12**	50%	Gibco, Thermo Fisher, 31331028
**Neurobasal**	50%	Gibco, Thermo Fisher, 21103049
**N2**	0.5x	Gibco, Thermo Fisher, 17502048
**B27**	0.5x	Gibco, Thermo Fisher, 17504044
**P/S**	1x	Gibco, Thermo Fisher, 15140122
**L-glut**	0.5x	Gibco, Thermo Fisher, 25030081
**NEAA**	0.5x	Gibco, Thermo Fisher, 11140050
**HEPES**	1x	Gibco, Thermo Fisher, 15630080
**2-Mercaptoetanol**	1x	Gibco, Thermo Fisher, 31350010
**Insulin**	2.5 µg/mL	Sigma-Aldrich, I9278
**SB (10 mM)**	10 µM	Stemcell technologies, 72234
**LDN (10mM)**	1 µM	Stemcell technologies, 72147

Table 2. Rosettes expansion medium.

Reagent	Final concentration	Supplier and reference
**DMEM/F12**	50%	Gibco, Thermo Fisher, 31331028
**Neurobasal**	50%	Gibco, Thermo Fisher, 21103049
**N2**	0.5x	Gibco, Thermo Fisher, 17502048
**B27**	0.5x	Gibco, Thermo Fisher, 17504044
**P/S**	1x	Gibco, Thermo Fisher, 15140122
**L-glut**	1 mM	Gibco, Thermo Fisher, 25030081
**NEAA**	0.5x	Gibco, Thermo Fisher, 11140050
**HEPES**	7.5 mM	Gibco, Thermo Fisher, 15630080
**2-Mercaptoetanol**	50 µM	Gibco, Thermo Fisher, 31350010
**Insulin**	2.5 µg/mL	Sigma-Aldrich, I9278
**bFGF (10 µg/mL)**	20 ng/mL	Peprotech, 100-18B

Table 3. Neural maintenance medium.

Reagent	Final concentration	Supplier and reference
**DMEM/F12**	50%	Gibco, Thermo Fisher, 31331028
**Neurobasal**	50%	Gibco, Thermo Fisher, 21103049
**N2**	0.5x	Gibco, Thermo Fisher, 17502048
**B27**	0.5x	Gibco, Thermo Fisher, 17504044
**P/S**	1x	Gibco, Thermo Fisher, 15140122
**L-glut**	1 mM	Gibco, Thermo Fisher, 25030081
**NEAA**	0.5x	Gibco, Thermo Fisher, 11140050
**HEPES**	7.5 mM	Gibco, Thermo Fisher, 15630080
**2-Mercaptoetanol**	50 μM	Gibco, Thermo Fisher, 31350010
**Insulin**	2.5 μg/mL	Sigma-Aldrich, I9278

Table 4. Glial expansion medium.

Reagent	Final concentration	Supplier and reference
**DMEM/F12**		Gibco, Thermo Fisher, 31331028
**N2**	1x	Gibco, Thermo Fisher, 17502048
**B27**	1x	Gibco, Thermo Fisher, 17504044
**P/S**	1x	Gibco, Thermo Fisher, 15140122
**NEAA**	1x	Gibco, Thermo Fisher, 11140050
**2-Mercaptoetanol**	50 μM	Gibco, Thermo Fisher, 31350010
**Insulin**	2.5 μg/mL	Sigma-Aldrich, I9278
**bFGF (10 μg/mL)**	10 ng/mL	Peprotech, 100-18B
**EGF (10 μg/mL)**	10 ng/mL	Peprotech, 100-15

Table 5. Astrocyte induction medium.

Reagent	Final concentration	Supplier and reference
**DMEM/F12**		Gibco, Thermo Fisher, 31331028
**N2**	1x	Gibco, Thermo Fisher, 17502048
**B27**	1x	Gibco, Thermo Fisher, 17504044
**P/S**	1x	Gibco, Thermo Fisher, 15140122
**NEAA**	1x	Gibco, Thermo Fisher, 11140050
**2-Mercaptoetanol**	50 μM	Gibco, Thermo Fisher, 31350010

**Insulin**	2.5 µg/mL	Sigma-Aldrich, I9278
**LIF (10 µg/mL)**	10 ng/mL	Peprotech, 300-05
**EFG (10 µg/mL)**	10 ng/mL	Peprotech 100-15

Table 6. Astrocyte maturation medium.

Reagent	Final concentration	Supplier and reference
**DMEM/F12**		Gibco, Thermo Fisher, 31331028
**N2**	1x	Gibco, Thermo Fisher, 17502048
**B27**	1x	Gibco, Thermo Fisher, 17504044
**P/S**	1x	Gibco, Thermo Fisher, 15140122
**NEAA**	1x	Gibco, Thermo Fisher, 11140050
**2-Mercaptoetanol**	50 µM	Gibco, Thermo Fisher, 31350010
**Insulin**	2.5 µg/mL	Sigma-Aldrich, I9278
**CNTF (10 µg/mL)**	20 ng/mL	Peprotech, 450-13

Table 7. Co-culture medium.

Reagent	Final concentration	Supplier and reference
**DMEM/F12**	25%	Gibco, Thermo Fisher, 31331028
**Neurobasal**	25%	Gibco, Thermo Fisher, 21103049
**Brainphys**	50%	Stemcell technologies, 05790
**N2**	0.5x	Gibco, Thermo Fisher, 17502048
**B27**	0.5x	Gibco, Thermo Fisher, 17504044
**P/S**	1x	Gibco, Thermo Fisher, 15140122
**L-glut**	1 mM	Gibco, Thermo Fisher, 25030081
**NEAA**	0.5x	Gibco, Thermo Fisher, 11140050
**Hepes**	7.5 mM	Gibco, Thermo Fisher, 15630080
**2-Mercaptoetanol**	50 µM	Gibco, Thermo Fisher, 31350010
**Insulin**	2.5 µg/mL	Sigma-Aldrich, I9278
**dbcAMP (10 mM)**	1 µM	Sigma-Aldrich, D0260

Table 8. Antibodies related to immunocytochemistry and flow cytometry.

Antibody	Dilution	Host	Supplier and reference
**Anti-ALDH1L1**	1:50	Rabbit	Proteintech, 17390-1-AP
**Anti-AQP4**	1:500	Rabbit	Sigma Aldrich, A5971
**Anti-β3 tubulin**	1:500	Rabbit	Synaptic Systems, 302 302
**Anti-GLAST**	1:200	Rabbit	Abcam, ab416
**Anti-GFAP**	1:500	Rabbit	Dako, z0334
**Anti-GFAP**	1:2000	Chicken	Millipore, AB5541
**Anti-Phospho-Histone H2A.X**	1:200	Rabbit	Cell Signaling, 2577S
**Anti-Ki67**	1:200	Mouse	BD Pharmigen, 556003
**Anti-LC3B**	1:100	Rabbit	Cell signaling, 27755
**Anti-MAP2**	1:200	Rabbit	Millipore, AB5622
**Anti-MAP2**	1:200	Mouse	Millipore, MAB3418
**Anti-PSD95**	1:250	Goat	Abcam, ab12093
**Anti-P21**	1:400	Rabbit	Abcam, ab188224
**Anti-Synaptophysin**	1:200	Rabbit	Abcam, 14692
**Anti-TOMM20**	1:200	Mouse	Abcam, AB56783
**Anti-Vimentin**	1:5,5	Mouse	DSHB, 3CB2-s

Table 9. Secondary antibodies used for immunostainings and flow cytometry.

Antibody	Fluorochrome	Dilution	Host	Supplier and reference
**Anti-chicken IgG**	Alexa Fluor 488	1:1000	Goat	Invitrogen, A11039
**Anti-rabbit IgG**	Alexa Fluor 488	1:500	Goat	Invitrogen, A11008
**Anti-rabbit IgG**	Alexa Fluor 568	1:500<br>1:1000	Goat	Invitrogen, A11011
**Anti-mouse IgG**	Alexa Fluor 488	1:500	Goat	Invitrogen, A10037
**Anti-mouse IgG**	Alexa Fluor 568	1:500	Goat	Invitrogen, A11004
**Anti-mouse IgM**	Alexa Fluor 568	1:500	Goat	Invitrogen, A21043
**Anti-mouse IgG**	Alexa Fluor 568	1:500	Donkey	Invitrogen, A10037
**Anti-goat IgG**	Alexa Fluor 488	1:500	Donkey	Invitrogen, A11055
**Anti-rabbit IgG**	Cy5	1:500	Donkey	Jackson Inmunoresearch, 711-175-152

Table 10. Antibodies related to western blot.

Antibody	Dilution	Host	Band	Supplier and reference
**Anti-pDrp1**	1:500	Rabbit	78 - 82 kDa	Cell signaling, 3455
**Anti-GAPDH**	1:3000	Mouse	36 kDa	Proteintech, 60004-1
**Anti-LAMP1**	1:2000	Rat	120, 90 kDa	Sigma-Aldrich, MABC39
**Anti-LAMP2**	1:200	Rat	120, 70, 50, 35 kDa	Santa Cruz ABL-93 sc 20004
**Anti-LC3B**	1:1000	Rabbit	15 kDa	Cell signaling, 2775S
**Anti-MFN2**	1:1000	Rabbit	80 kDa	Cell signaling, 9482S
**Anti-OPA1**	1:3000	Rabbit	80 - 90 kDa	Proteintech, 27733
**Anti-PARK2**	1:1000	Rabbit	70, 42 - 52 kDa	Proteintech, 14060-1-AP
**Anti-PINK1**	1:1000	Rabbit	65 - 45 kDa	Proteintech, 23274-1-AP
**Anti-PSD95**	1:1000	Rabbit	95 kDa	Abcam ab18258
**Anti-Synaptophysin**	1:500	Rabbit	42 kDa	Abcam ab14692

Table 11. Secondary antibodies used for western blot.

Antibody	Dilution	Host	Supplier and reference
**Anti-Rabbit Ig**	1:3000	Goat	Bio-Rad, 1706515
**Anti-Mouse Ig**	1:3000	Goat	Bio-Rad, 1706516
**Anti-Rat Ig**	1:3000	Rabbit	Dako, E0468

Table 12: List of primer sequences used for gene expression analysis by qPCR.

Gen	*Forward primer*	*Reverse primer*
**IL1α**	TGTATGTGACTGCCCAAGATGAAG	AGAGGAGGTTGGTCTCACTACC
**CXCL8**	GAGAGTGATTGAGAGTGGACCAC	CACAACCCTCTGCACCCAGTTT
**IGF1**	CTCTTCAGTTCGTGTGTGGAGAC	CAGCCTCCTTAGATCACAGCTC
**IGFBP4**	ACCCACGAGGACCTCTACATCA	CACACCAGCACTTGCCACGCT
**EGF**	TGCGATGCCAAGCAGTCTGTGA	GCATAGCCCAATCTGAGAACCAC
**EGFR**	AACACCCTGGTCTGGAAGTACG	TCGTTGGACAGCCTTCAAGACC
**MMP2**	AGCGAGTGGATGCCGCCTTTAA	CATTCCAGGCATCTGCGATGAG
**PLAT**	TGGTGCTACGTCTTTAAGGCGG	GCTGACCCATTCCCAAAGTAGC
**HGF**	GAGAGTTGGGTTCTTACTGCACG	CTCATCTCCTCTTCCGTGGACA
**NFKB1**	GCAGCACTACTTCTTGACCACC	TCTGCTCCTGAGCATTGACGTC
**GM-CSF**	GGAGCATGTGAATGCCATCCAG	CTGGAGGTCAAACATTTCTGAGAT
**IL6**	AGACAGCCACTCACCTCTTCAG	TTCTGCCAGTGCCTCTTTGCTG
**IL8**	GAGAGTGATTGAGAGTGGACCAC	CACAACCCTCTGCACCCAGTTT
**CCL2**	AGAATCACCAGCAGCAAGTGTCC	TCCTGAACCCACTTCTGCTTGG
**IL1β**	CCACAGACCTTCCAGGAGAATG	GTGCAGTTCAGTGATCGTACAGG
**TNFα**	CTCTTCTGCCTGCTGCACTTTG	ATGGGCTACAGGCTTGTCACTC
**IL12A**	TGCCTTCACCACTCCCAAAACC	CAATCTCTTCAGAAGTGCAAGGG
**IFNγ**	GAGTGTGGAGACCATCAAGGAAG	TGCTTTGCGTTGGACATTCAAGTC
**IL23A**	GAGCCTTCTCTGCTCCCTGATA	GACTGAGGCTTGGAATCTGCTG
**IL10**	TCTCCGAGATGCCTTCAGCAGA	TCAGACAAGGCTTGGCAACCCA
**CXCL10**	GGTGAGAAGAGATGTCTGAATCC	GTCCATCCTTGGAAGCACTGCA
**CCL3**	ACTTTGAGACGAGCAGCCAGTG	TTTCTGGACCCACTCCTCACTG
**CCL5**	CCTGCTGCTTTGCCTACATTGC	ACACACTTGGCGGTTCTTTCGG
